# Defining E3 ligase–substrate relationships through multiplex CRISPR screening

**DOI:** 10.1038/s41556-023-01229-2

**Published:** 2023-09-21

**Authors:** Richard T. Timms, Elijah L. Mena, Yumei Leng, Mamie Z. Li, Iva A. Tchasovnikarova, Itay Koren, Stephen J. Elledge

**Affiliations:** 1 Department of Genetics, Harvard Medical School, Division of Genetics, Brigham asnd Women’s Hospital, Howard Hughes Medical Institute, Boston, MA USA; 2 Cambridge Institute of Therapeutic Immunology and Infectious Disease, Department of Medicine, University of Cambridge, Cambridge, UK; 3 Wellcome/CRUK Gurdon Institute, Department of Biochemistry, University of Cambridge, Cambridge, UK; 4 The Mina and Everard Goodman Faculty of Life Sciences, Bar-Ilan University, Ramat-Gan, Israel

**Keywords:** High-throughput screening, Ubiquitin ligases, CRISPR-Cas9 genome editing

## Abstract

Specificity within the ubiquitin–proteasome system is primarily achieved through E3 ubiquitin ligases, but for many E3s their substrates—and in particular the molecular features (degrons) that they recognize—remain largely unknown. Current approaches for assigning E3s to their cognate substrates are tedious and low throughput. Here we developed a multiplex CRISPR screening platform to assign E3 ligases to their cognate substrates at scale. A proof-of-principle multiplex screen successfully performed ~100 CRISPR screens in a single experiment, refining known C-degron pathways and identifying an additional pathway through which Cul2^FEM1B^ targets C-terminal proline. Further, by identifying substrates for Cul1^FBXO38^, Cul2^APPBP2^, Cul3^GAN^, Cul3^KLHL8^, Cul3^KLHL9/13^ and Cul3^KLHL15^, we demonstrate that the approach is compatible with pools of full-length protein substrates of varying stabilities and, when combined with site-saturation mutagenesis, can assign E3 ligases to their cognate degron motifs. Thus, multiplex CRISPR screening will accelerate our understanding of how specificity is achieved within the ubiquitin–proteasome system.

## Abstract (web summary)

Timms, Mena et al. present a multiplex CRISPR screening platform capable of assigning E3 ligases to their substrates and degrons at scale.

## Main

The degradation of intracellular proteins plays a central role in the regulation of a myriad of cellular processes^1^. The ubiquitin–proteasome system (UPS) is one of the primary routes through which the cell achieves selective protein degradation, wherein proteins are tagged with ubiquitin that signals for their degradation by the proteasome. Typically, E3 ubiquitin ligases directly recognize protein substrates for ubiquitylation and are thus the primary determinants of specificity within the UPS. This is thought to be achieved largely through their ability to selectively recognize specific molecular features of their substrates, which are known as degrons. Although our knowledge remains sparse, the majority of known degrons comprise short linear motifs lying in accessible regions of proteins^2^. Degrons can either act constitutively, promoting continuous degradation of the protein, or conditionally, allowing protein turnover to be regulated through post-translational modifications such as phosphorylation^3^.

The human genome encodes >600 E3 ubiquitin ligases, which act post-translationally to regulate the activity and stability of the entire proteome^4^. Given this vast complexity, one of the central challenges in the field is the identification of UPS substrates and delineation of their cognate E3 ligases; indeed, for many E3s their substrates remain unknown. Proteomic techniques have traditionally been used to define the substrates of E3 ligases, but these remain labour intensive and low throughput and, in the case of co-immunoprecipitation approaches, may fail to detect transient interactions^5^. We have pioneered a genetic approach called Global Protein Stability (GPS)^6^, which allows for the simultaneous stability profiling of pools of thousands of substrates. GPS is a lentiviral platform in which libraries of either short peptides or full-length open reading frames (ORFs) are fused to green fluorescent protein (GFP). Upon expression in human cells, the relative expression of the GFP-fusion protein relative to a DsRed internal control expressed from the same construct can be used to infer the stability (that is, the lifetime in cells) of the fusion protein. In a library format, cells are sorted using fluorescence-activated cell sorting (FACS) into a series of bins based on the stability of the fusion proteins, which can then be deconvoluted by next-generation sequencing to yield a stability profile for each individual substrate. The GPS system has been used by us and others to identify substrates of Cullin-RING ligases (CRLs)^7,8^, targets of molecular glues^9^, quality control substrates^10^, N-terminal degrons^11^ and C-terminal degrons^12^. However, despite its power in identifying UPS substrates, assigning the E3 ligase responsible requires a clustered regularly interspaced short palindromic repeats (CRISPR) screen to be performed on each individual GFP-fusion substrate. The need to perform CRISPR screens individually severely limits the throughput of the approach, as realistically only a handful of substrates can be characterized in this manner at once.

In this Technical Report, we developed a multiplexed CRISPR screening platform that allows the simultaneous mapping of E3 ligases to hundreds of substrates in parallel. We demonstrate its utility by performing multiplexed CRISPR screens using substrate libraries comprising both short peptides and full-length protein substrates, and we map individual degron motifs using site-saturation mutagenesis.

## Results

### Design of a multiplex CRISPR screening platform

**Fig. 1:**
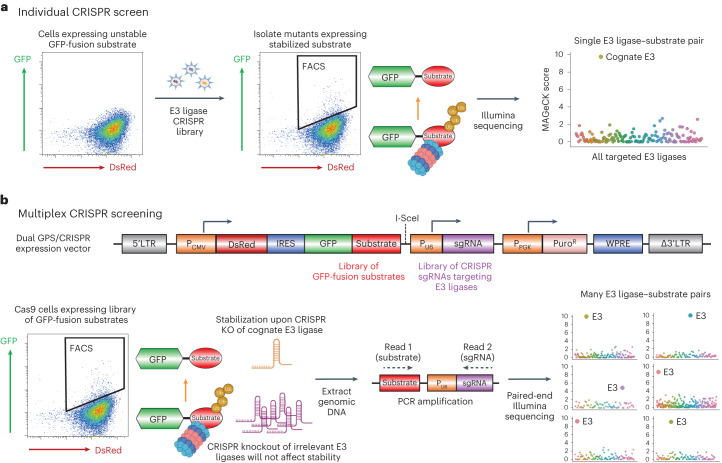
Design of the multiplex CRISPR screening platform. **a**, Individual FACS-based CRISPR screens are highly effective at identifying the cognate E3 ligase for unstable substrates tagged with a fluorescent protein such as GFP, but suffer from limited throughput as they are only capable of analysing a single substrate per screen. **b**, In contrast, multiplex CRISPR screening aims to identify the cognate E3 ligases for tens or hundreds of substrates in a single experiment. By encoding a library of GFP-tagged substrates and CRISPR sgRNAs targeting E3 ligases on the same lentiviral vector, cells expressing stabilized substrates paired with an sgRNA targeting the cognate E3 ligase can be enriched by FACS and the combination identified by paired-end sequencing. LTR, long terminal repeat; P_CMV_, human cytomegalovirus promoter; IRES, internal ribosome entry site; P_PGK_, phosphoglycerate kinase promoter; WPRE, Woodchuck Hepatitis Virus post-transcriptional regulatory element.

CRISPR screens represent a powerful approach for assigning E3 ubiquitin ligases to their cognate substrates. Typically, cells expressing an unstable substrate tagged with GFP are transduced with Cas9 and a library of CRISPR single guide RNAs (sgRNAs) targeting, for example, all known E3 ubiquitin ligases (for instance, ref. ^11^). CRISPR-mediated disruption of the cognate E3 ligase will result in stabilization of the substrate and hence an increase in GFP fluorescence; these cells can be isolated by FACS and the identity of the guide RNAs enriched in these cells determined by polymerase chain reaction (PCR) amplification followed by Illumina sequencing (Fig. 1a). This approach has proven extremely successful across many laboratories, but is fundamentally limited in scale as only one substrate can be assayed per screen. Thus, we set out to adapt this approach to develop a platform that would permit high-throughput identification of E3 ligase substrates.

Our multiplex CRISPR screening approach combines the GPS expression screening technique with loss-of-function CRISPR screens to identify the E3 ligases responsible for the instability of GFP-fusion proteins. We reasoned that we could perform many CRISPR screens in parallel by encoding both the GFP-tagged substrates and the CRISPR sgRNAs together on the same vector. Starting with a standard GPS lentiviral expression vector, we first cloned a library of substrates as C-terminal fusions to GFP; subsequently we cloned in a library of CRISPR sgRNAs driven by the U6 promoter (Fig. 1b). Following transduction of Cas9-expressing target cells at low multiplicity of infection and puromycin selection to eliminate untransduced cells, each cell in the resulting population expresses one GFP-tagged substrate and one sgRNA targeting an E3 ubiquitin ligase. In the vast majority of cells, the sgRNA will target an irrelevant E3 ligase that will not affect the stability of the GFP-fusion protein; however, in rare cells the sgRNA will disrupt the cognate E3 ligase, resulting in stabilization of the fusion protein and an increase in GFP fluorescence. Cells expressing stabilised substrates can be isolated by FACS, followed by PCR amplification and paired-end sequencing to identify the GFP-fusion substrate (forward read) together with the E3 ligase targeted by the sgRNA (reverse read) (Fig. 1b). The identity of peptide substrates is revealed by directly sequencing the nucleotides that encode them, whereas full-length proteins are identified by sequencing an associated DNA barcode located at their 3′ end.

### A proof-of-principle multiplex CRISPR screen

**Fig. 2:**
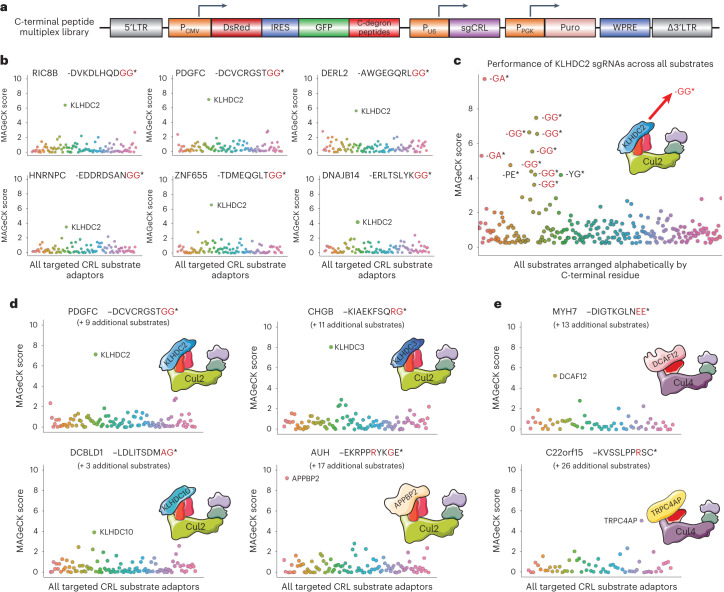
A proof-of-principle multiplex CRISPR screen recapitulates known C-degron pathways. **a**, Schematic representation of the dual GPS/CRISPR multiplex screening library, in which the GFP-fusion substrates were a pool of peptides enriched for C-terminal degrons targeted by Cul2 or Cul4 E3 ligase complexes, and the CRISPR sgRNA library targeted either Cul2/5 or Cul4 adaptors. **b**,**c**, Identification of KLHDC2 substrates bearing C-terminal di-glycine motifs: the multiplex screen results for six example substrates, all of which terminate with two glycine residues (**b**); the performance of sgRNAs targeting KLHDC2 across all substrates (**c**). **d**,**e**, Cullin adaptors are correctly assigned to their cognate C-terminal degrons. A range of peptide substrates bearing canonical C-degron motifs targeted by Cul2 (**d**) and Cul4 (**e**) adaptors were successfully identified. All source numerical data are available in Supplementary Tables 1–6.

**Extended Data Fig. 1:**
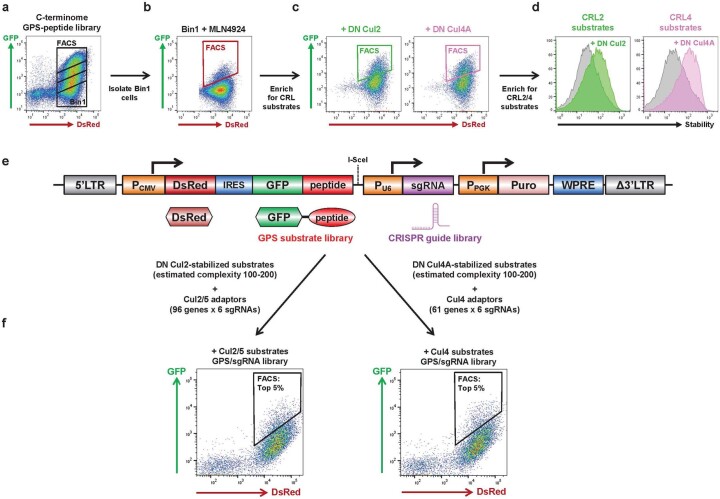
Generation of multiplex CRISPR screen libraries to interrogate C-terminal degrons targeted by Cullin-RING E3 ligases. **a-d** Derivation of cells expressing C-terminal peptides targeted by Cul2 complex or Cul4 complexes. Starting from HEK-293T cells expressing the C-terminome GPS-peptide library^12^, we isolated the most unstable GFP-peptide fusions (Bin1) **(a)**, treated them with MLN4924, and then isolated cells in which the GFP-peptides fusions were stabilized **(b)**. After recovery in the absence of MLN4924, to further purify substrates of specific Cullin complexes we expressed dominant-negative (DN) versions of either Cul2 (green) or Cul4A (pink) and again isolated the cells in which the GFP-peptides fusions were stabilized by FACS **(c)**. After recovery, we re-challenged the sorted cells with the DN Cullins to verify that the final populations of cells were highly enriched for CRL substrates **(d)**. **e,f**, Generation of the multiplex CRISPR screening vector to examine C-terminal degrons targeted by Cullins. **e**, Schematic representation of the multiplex CRISPR screening vector. GFP-fusion peptides were amplified from the genomic DNA of the cells in (d) by PCR and cloned into the lentiviral vector downstream of GFP; the CRISPR sgRNA library targeting either Cul2 or Cul4 adaptors was then cloned into the resulting substrate library using the I-SceI site to generate the dual GPS-sgRNA multiplex CRISPR screening library. **f**, The library was then introduced into HEK-293T stably expressing Cas9, and the top ~5% of cells expressing the most stable substrates were isolated by FACS. Genomic DNA was then extracted from both the sorted cells and the unsorted libraries, and substrate-sgRNA pairs enriched in the sorted cells quantified by paired-end Illumina sequencing.

To validate that our platform was capable of successfully performing many simultaneous CRISPR screens, we leveraged our previous findings delineating C-terminal degron pathways^12^ to design a proof-of-principle screen. Previously we generated pools of cells expressing GPS constructs in which 23-mer peptides derived from the C-termini of human proteins were fused to GFP and used FACS to isolate cells expressing GFP–peptide fusions that were stabilized upon expression of dominant-negative (DN) versions of Cul2 and Cul4 (ref. ^12^) (Extended Data Fig. 1a–d). We extracted genomic DNA from these cells, PCR-amplified the peptides encoded by the lentiviral GPS construct, and cloned the resulting pool of PCR products into the GPS vector. To create the dual GPS/CRISPR vector for multiplex screening, we subsequently cloned in an sgRNA expression cassette comprising a library of guides targeting either all known Cul2/5 substrate adaptors (96 genes) or Cul4A/4B substrate adaptors (61 genes) (Fig. 2a and Extended Data Fig. 1e). We estimated that the complexity of the substrate library was ~100 peptides in each case, resulting in a matrix of ~100 peptides × 96 or 61 genes × 6 sgRNAs/gene = ~50,000 substrate–guide combinations. We isolated the top ~5% of cells on the basis of the stability of the GFP–peptide fusion (Extended Data Fig. 1f), amplified and sequenced the lentiviral constructs, and then used the MAGeCK algorithm^13^ to identify substrate–guide RNA combinations enriched in the selected cells versus the unsorted starting population (Supplementary Table 1). We aimed to maintain at least 100-fold representation at each step, resulting in a total of ~5 million sorted cells.

As a result of our previous work on C-terminal degron pathways^12^, a large number of known CRL adaptor–degron pairs served as positive controls. Overwhelmingly, substrates bearing known C-terminal degrons were correctly assigned to their cognate adaptor (Fig. 2b–e). KLHDC2, for example, was identified as a significant hit for 11 peptide substrates, the screen results for 6 of which are depicted in Fig. 2b. Seven of these terminated with -GG*, the canonical KLHDC2 C-degron, and two terminated with the highly similar motif -GA* (Fig. 2c). Analogous results were obtained for a variety of other Cul2 adaptors known to target C-terminal degrons (Supplementary Tables 1–3): 12 KLHDC3 substrates and 4 KLHDC10 substrates respectively terminated with glycine residues, while 18 APPBP2 substrates harboured RxxG motifs near their C-terminus (one representative substrate for each is shown in Fig. 2d). In parallel, the Cul4 screen revealed a large number of substrates bearing the canonical C-degron -EE* and -Rxx* motifs targeted by DCAF12 and TRPC4AP, respectively (Fig. 2e and Supplementary Tables 4–6). Altogether, we estimate that we performed ~100 successful CRISPR screens in parallel.

### FEM1B targets C-terminal proline

**Fig. 3:**
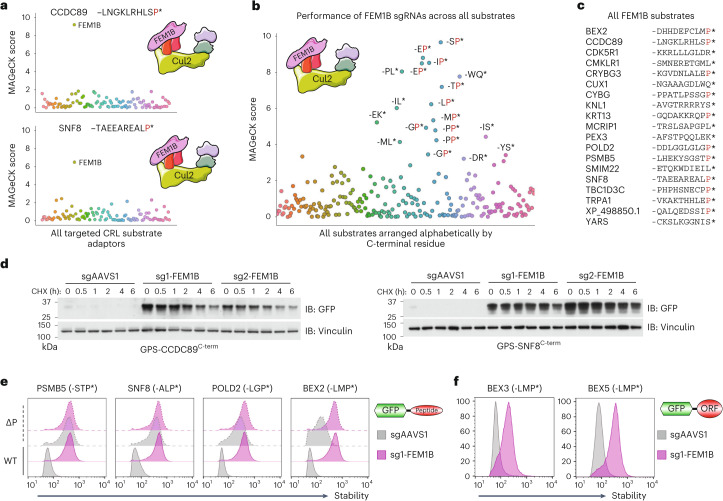
Cul2^FEM1B^ regulates a C-degron pathway specific for proline. **a**–**c**, FEM1B substrates are highly enriched for C-terminal proline: screen results for two example substrates (**a**), the performance of sgRNAs targeting FEM1B across all substrates (**b**) and a tabulation of the sequences of all substrates for which FEM1B was a significant hit (**c**), with terminal proline residues indicated in red. **d**, Cycloheximide chase assays to monitor the degradation of the indicated GPS substrates in control (sgAAVS1) or FEM1B knockout (sgFEM1B) cells by immunoblot (IB). **e**,**f**, FEM1B targets C-terminal proline: C-terminal 23-mer peptides derived from the indicated genes, either with (wild type, WT) or without (ΔP) their terminal proline residue, were expressed in control (sgAAVS1) and FEM1B knockout (sgFEM1B) cells in the context of the GPS system and their stability measured by flow cytometry (**e**); full-length ORFs of the BEX family terminating in proline were more stable in FEM1B knockout cells (**f**). Immunoblot and flow cytometry experiments were performed twice with similar results. All source numerical data are available in Supplementary Tables 1–6; unprocessed blots are available in source data. Source data

**Extended Data Fig. 2:**
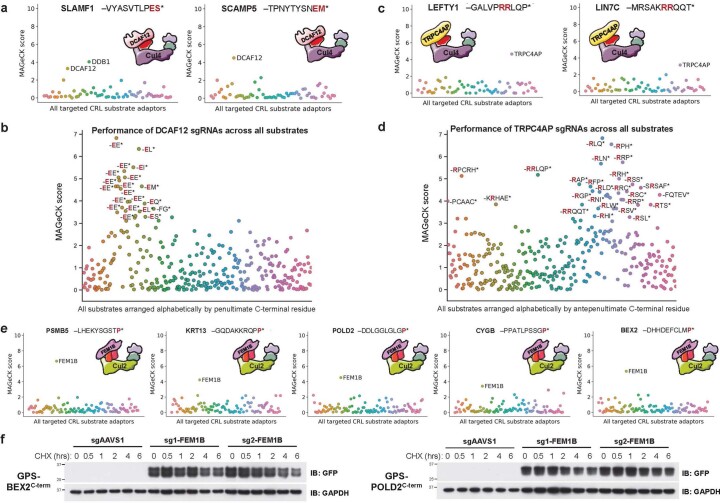
Novel aspects of C-degron pathways revealed by the multiplex CRISPR screen. **a-d**, DCAF12 recognizes a wider variety of motifs characterized by a glutamic acid at the penultimate position (**a,b**), while TRPC4AP can recognize substrates with arginine residues at the -4 and -5 position as well as those with arginine at -3 (**c,d**). **e**, Peptide substrates of FEM1B are enriched for C-terminal proline. Screen results for six substrates in which FEM1B was identified as a significant hit are shown; inspection of the sequences of the peptides revealed that they all terminate with a C-terminal proline residue (highlighted in red). **f**, Cycloheximide chase assays to monitor the degradation of the indicated GPS substrates in control (sgAAVS1) or FEM1B knockout (sgFEM1B) cells by immunoblot. Immunoblot experiments were performed twice with similar results. All source numerical data are available in Supplementary Tables 1-6; unprocessed blots are available in source data. Source data

Due to the breadth of our multiplexing approach, not only did our screen recapitulate known C-degron pathways, but it also revealed additional insights. First, we uncovered an expanded repertoire of C-terminal degrons targeted by Cul4^DCAF12^ and Cul4^TRPC4AP^. In addition to terminal -EE* motifs, we found a significant number of DCAF12 substrates that comprised a glutamic acid at the penultimate position but harboured non-glutamic acid residues at their C-terminus, with substrates terminating in -EI*, -EM* and -ES* (Extended Data Fig. 2a,b). Thus, the most critical part of the C-terminal degron recognized by DCAF12 is the glutamic acid at the −2 position, which is consistent with a recent proteomic analysis of DCAF12 substrates^14^. Similarly, our previous definition of the TRPC4AP degron as an R-3 motif is too rigid; several of the TRPC4AP degrons identified did not contain an arginine at the −3 position, but instead harboured arginine residues at the −4 and/or −5 positions (Extended Data Fig. 2c,d). Most significantly, however, we uncovered a large number of substrates targeted by FEM1B (Fig. 3a,b and Extended Data Fig. 2e), a Cul2 adaptor known to participate in C-degron recognition but for which a degron motif is not currently well defined. Intriguingly, we noted that the majority of FEM1B substrates terminated with a proline residue (Fig. 3b,c).

To validate that FEM1B does indeed regulate a C-terminal degron pathway specific for proline residues, we performed individual validation experiments using a panel of example C-terminal peptides fused to GFP. In support of the multiplex CRISPR screening results, we found that all of the substrates were indeed stabilized upon ablation of FEM1B (Fig. 3d and Extended Data Fig. 2f); importantly, this effect required the C-terminal proline residue (Fig. 3e). Furthermore, our GPS-ORFeome screens (see below) identified full-length proteins of the BEX family as Cul2 substrates. As BEX proteins all terminate with C-terminal proline, we hypothesized that they would be targeted by FEM1B, which we confirmed for BEX3 and BEX5 expressed in the context of the GPS system (Fig. 3f). Interestingly, the BEX proteins have been recently described as pseudosubstrates of FEM1B that regulates its activity in the reductive stress response pathway^15^, highlighting the utility of our approach in identifying important pathways. Thus, multiplex CRISPR screening uncovered a Pro/C-degron pathway regulated by Cul2^FEM1B^.

### FEM1B uses multiple sites to recognize diverse degrons

**Fig. 4:**
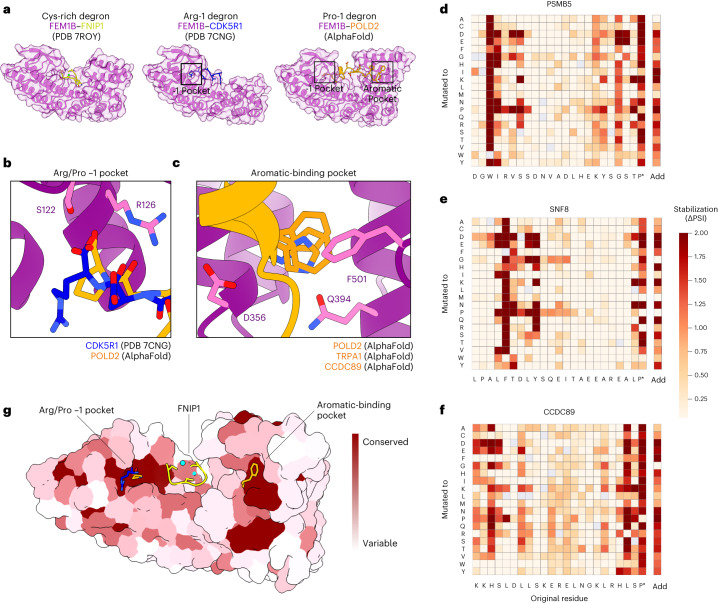
FEM1B uses multiple pockets to bind diverse degrons. **a**–**c**, Structural analysis of FEM1B–degron interactions: overview of existing structures of FEM1B (purple) bound to the cysteine-rich substrate FNIP1 (yellow) or the Arg-ended CDK5R1 C-terminus (blue), compared with AlphaFold predictions of FEM1B bound to a representative Pro-end degron (POLD2, orange) (**a**); the Arg/Pro -1 pocket of FEM1B (purple) is shown bound to the CDK5R1 Arg-end substrate (blue) and the POLD2 Pro-end substrate (orange) (**b**); the aromatic-binding pocket of FEM1B (purple) is shown bound to three substrates (orange) that each requires a Phe, Trp or His to be recognized by FEM1B (**c**). **d**–**f**, Pro-ended FEM1B substrates require a hydrophobic residue ~15–20 residues from the C-terminus for efficient degradation. Saturation mutagenesis results for three representative Pro-ended substrates are shown, the C-terminus of PSMB5 (**d**), the C-terminus of SNF8 (**e**) and the C-terminus of CCDC89 (**f**); the darker the red colour, the greater the stabilizing effect of the mutation. The Add column indicates the effect of appending each individual amino acid at the extreme C-terminus of the peptide substrate. **g**, Evolutionary conservation of FEM1B binding pockets. The surface of FEM1B (residues 86–400) is coloured by conservation on the basis of an alignment of sequences from 12 diverse animal species. The sites for binding the Pro (orange) and Arg (blue) C-termini, FNIP1 (yellow) and zinc ions (cyan), and aromatic residues (orange) are shown for reference. Source numerical data are available in Supplementary Table 7.

**Extended Data Fig. 3:**
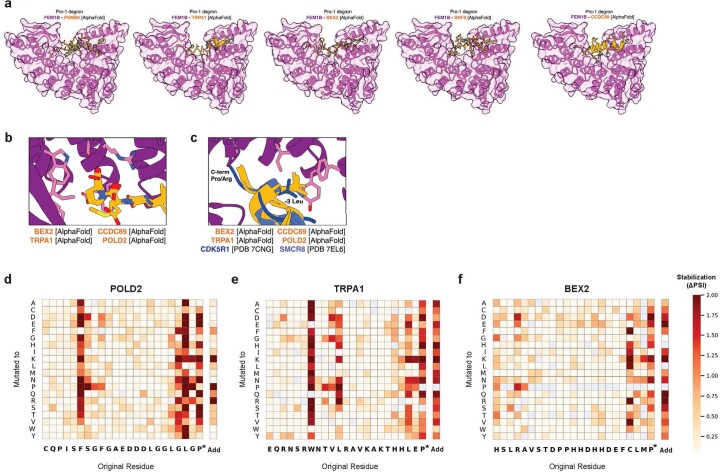
Analysis of FEM1B degrons. **a**, Additional AlphaFold structural predictions for the interactions between FEM1B and Pro-ended peptide substrates. **b**, The Arg/Pro -1 pocket of FEM1B is shown bound to four different Pro-ended degrons predicted to make similar interactions with FEM1B. **c**, Many of the Pro-end (orange) or Arg-end (blue) substrates use a leucine at the -3 position to interact with a surface on FEM1B (purple). **d-f**, Saturation mutagenesis results for additional Pro-ended substrates. Source numerical data are available in Supplementary Table 7.

As FEM1B has previously been shown to recognize C-terminal arginine degrons^12,16–18^ and an internal cysteine-rich sequence^15^, we were intrigued by its ability to target three seemingly distinct degrons. Thus, we used AlphaFold to predict the mode of interaction of FEM1B with C-terminal proline degrons and compared these predictions to existing FEM1B-substrate co-crystal structures^16–18^ (Fig. 4a and Extended Data Fig. 3a). AlphaFold2 predicted that the C-terminal proline substrates bind a deep pocket in FEM1B (Extended Data Fig. 3b). The proline side chain interacts with several hydrophobic residues lining the FEM1B pocket, while the C-terminal carboxylic acid of proline makes hydrogen bonds with Ser122 and Arg126 of FEM1B. This interaction is very similar to the interaction that FEM1B makes with C-terminal arginine substrates (Fig. 4a,b), suggesting that this “−1 pocket” can accommodate both proline and arginine C-terminal residues. Furthermore, both classes of degron often contain leucine at the −3 position, which binds to a nearby site on FEM1B (Extended Data Fig. 3c).

**Extended Data Fig. 4:**
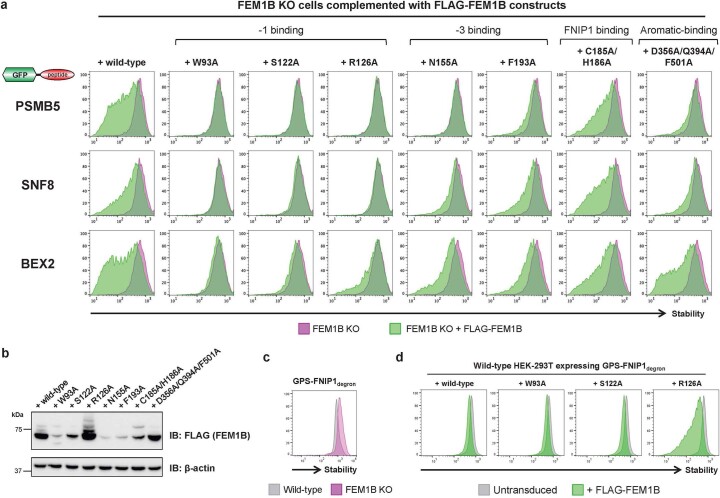
Genetic complementation of FEM1B knockout cells. **a-b**, Assessing the role of the predicted FEM1B binding pockets in the recognition of three peptide substrates terminating with proline. Three example GFP-tagged peptide substrates were selected: the C-termini of PSMB5 and SNF8, predicted by both AlphaFold and the saturation mutagenesis data to make essential contacts with both the Arg/Pro -1 pocket and the aromatic-binding pocket (Extended Data Fig. 3a,d,e), and the C-terminus of BEX2, which AlphaFold suggested also binds both pockets but for which the saturation mutagenesis suggested only the contacts with the Arg/Pro -1 pocket were essential for efficient degradation (Extended Data Fig. 3a,f). FEM1B knockout cells were first transduced with lentiviral vectors encoding FLAG-tagged wild-type FEM1B or FEM1B variants harboring the indicated mutations. Subsequently the cells were transduced with GPS vectors encoding the indicated peptide substrates of FEM1B terminating with C-terminal proline and their stability measured by flow cytometry **(a)**. Relative expression levels of the exogenous FEM1B constructs were assayed by immunoblot **(b)**. We attempted to validate that the -1 binding pocket mutants were competent for the degradation of GFP fused to a peptide degron from FNIP1 (ref. ^30^), but we found that the FNIP1_degron_ peptide was only minimally stabilized in FEM1B knockout cells when expressed in the context of the GPS system **(c)**. However, overexpression of FEM1B harboring the R126A mutation did result in substantial destabilization of the FNIP1 construct in wild-type cells **(d)**. Immunoblot and flow cytometry experiments were performed twice with similar results. Unprocessed blots are available in source data. Source data

Intriguingly, the AlphaFold predictions also suggested that a hydrophobic residue in the Pro-end peptide substrates bound a distinct site on FEM1B (Fig. 4a and Extended Data Fig. 3a). This residue is located approximately 15–20 residues before the C-terminal proline. Its side chain buries into an “aromatic-binding pocket” on the concave surface of FEM1B, bound by hydrophobic residues lining the interior of the pocket plus two glutamines on the outside of the pocket (Fig. 4c). We tested these predictions by performing saturation mutagenesis on several Pro-ended substrates predicted to engage both pockets (Supplementary Table 7). This revealed that both the C-terminal proline and an internal aromatic residue were generally required for efficient degradation (Fig. 4d–f and Extended Data Fig. 3d), supporting the structural models. In most cases the addition of any single amino acid at the C-terminus abrogated degradation, demonstrating the importance of the proline residue being positioned at the extreme C-terminus. Genetic complementation experiments in FEM1B knockout cells also supported the structural models (Extended Data Fig. 4).

Thus, Pro-end substrates are predicted to bind FEM1B using two sites: the −1 pocket of FEM1B binds the C-terminal proline, while the aromatic pocket binds an aromatic residue approximately 35 Å away. We note that a distinct region of FEM1B binds the cysteine-rich degron of FNIP1 via the joint coordination of two zinc ions^15^ (Fig. 4a,g). Therefore, FEM1B appears to have at least three separate regions for recognizing a variety of degrons, each bound in unique ways. Interestingly, the Arg/Pro −1 pocket and the aromatic-binding pocket are the most conserved evolutionarily (Fig. 4g).

### Multiplex CRISPR screens assign full-length substrates

**Fig. 5:**
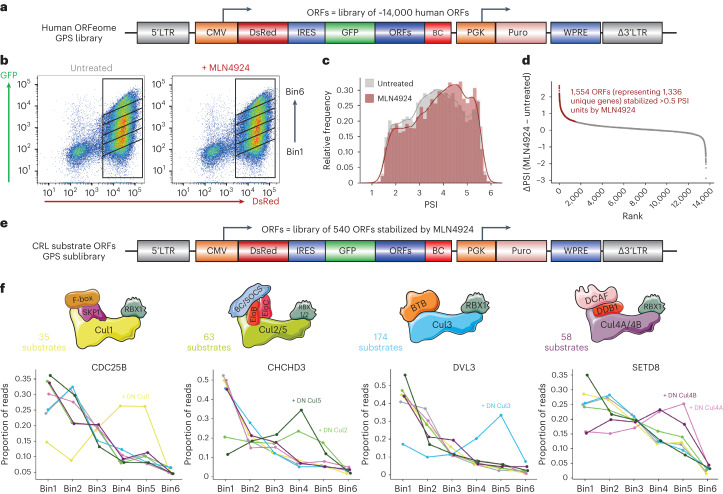
Stability profiling of the human ORFeome identifies substrates of Cullin-RING E3 ubiquitin ligases. **a**–**d**, Identifying substrates of Cullin-RING E3 ligases: schematic representation of the GPS ORFeome library, comprising approximately 14,000 full-length, sequence-verified barcoded human ORFs (**a**); schematic representation of the comparative stability profiling screen using the pan-Cullin inhibitor MLN4924, where the human ORFeome library was expressed in HEK-293T cells and partitioned into six equal bins by FACS, and using the same settings and gates, the process was repeated for cells treated with MLN4924 (**b**); overall distribution of stability scores, comparing untreated (grey) and MLN4924-treated (red) cells (**c**); and 1,554 ORFs exhibited stabilization >0.5 PSI units following MLN4924 treatment (**d**). **e**,**f**, Assigning substrates to individual Cullin complexes: schematic representation of the barcoded sublibrary comprising the top 540 ORFs exhibiting the greatest stabilization from **d** (**e**); comparative stability profiling was performed as depicted in **b** to assess the stability of the library in cells expressing either an empty vector (grey) versus C-terminally truncated DN versions of Cul1 (yellow), Cul2 (light green), Cul3 (light blue), Cul4A (pink), Cul4B (purple) or Cul5 (dark green) (**f**). Screen profiles for four example substrates are shown. Source numerical data are available in Supplementary Tables 8–12.

**Extended Data Fig. 5:**
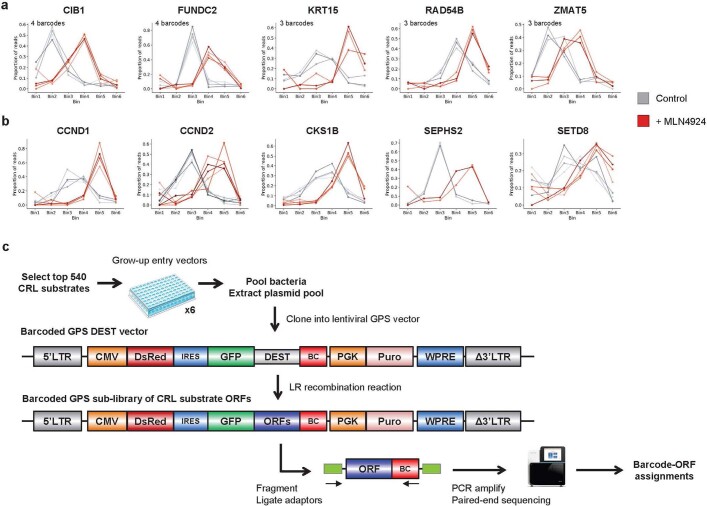
Proteome-wide stability profiling to identify substrates of Cullin-RING E3 ligases. **a**, The GPS-ORFeome screen exhibited high reproducibility. Example screen profiles for the indicated ORFs are shown, reflecting the distribution of sequencing reads across the 6 stability bins. Colored lines (grays, control; reds, MLN4924) represent different barcodes attached to the same ORF. **b**, The GPS-ORFeome screen identified known CRL substrates. Example screen profiles for a range of positive control substrates are shown. **c**, Generation of barcoded sub-library of ORFs stabilized by MLN4924. Bacteria harboring individual ORF constructs in Gateway entry vectors were grown up, pooled, and a Gateway LR reaction performed to shuttle the ORFs into a barcoded GPS lentiviral destination vector. Barcode-ORF pairs were subsequently assigned by paired-end Illumina sequencing.

Next, we set out to adapt the multiplex CRISPR screening platform to allow the identification of E3 ubiquitin ligases targeting full-length protein substrates. To generate a suitable pool of full-length protein substrates targeted by CRLs, we began by performing a GPS screen using the barcoded human ORFeome^12,19^ (Fig. 5a). Comparative stability profiling in the presence and absence of MLN4924 (Fig. 5b), a pan-CRL small molecule inhibitor^20^, identified ~1,500 ORFs as candidate CRL substrates in HEK-293T cells (Fig. 5c,d and Supplementary Tables 8–10). An advantage of this system is that each ORF is associated with on average approximately five unique barcodes, thereby providing internal replicates; we observed strong concordance between the stability profiles of each individual barcode associated with the same ORF (Extended Data Fig. 5a). Furthermore, we identified a range of known CRL substrates as positive controls (Extended Data Fig. 5b).

Subsequently we focused on the top 540 ORFs that exhibited the greatest degree of stabilization upon MLN4924 treatment. To identify which Cullin complex was responsible for their degradation, we generated a barcoded sublibrary containing these 540 ORFs (Extended Data Fig. 5c) and performed a further GPS assay to compare their stability in cells transduced with an empty vector versus those expressing DN versions of Cul1, Cul2, Cul3, Cul4A, Cul4B and Cul5 (Fig. 5e and Supplementary Table 4). This assigned ~60% of the substrates to either Cul1, Cul2/5, Cul3 or Cul4A/4B complexes (Supplementary Tables 11 and 12); example profiles for positive control substrates are shown in Fig. 5f. Thus, together these datasets represent a rich resource to guide further exploration of the substrate repertoire regulated by CRLs.

**Fig. 6:**
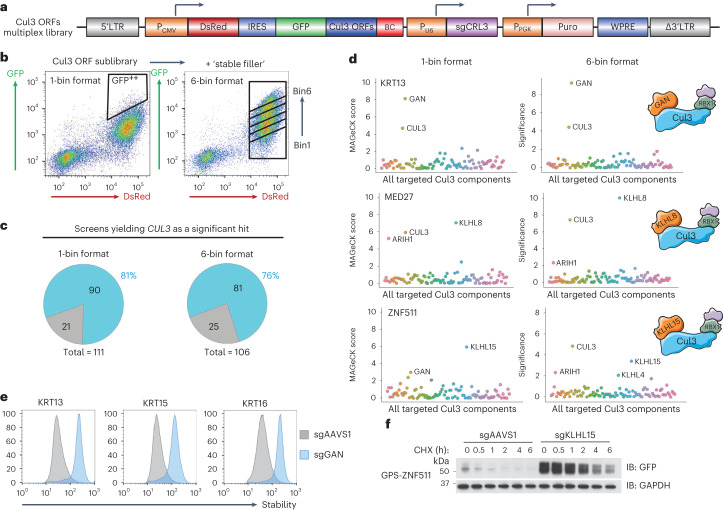
A multiplex CRISPR screen to identify the cognate adaptors required for full-length protein substrates targeted by Cul3 complexes. **a**, Schematic representation of the multiplex CRISPR screening vector, wherein ~100 full-length ORFs targeted by Cul3 complexes were fused to the C-terminus of GFP, and the CRISPR sgRNA library targeted known BTB adaptors. **b**, The multiplex CRISPR screen was performed in two ways: in the 1-bin format (left), the top ~5% of the population was sorted into a single bin, while in the 6-bin format (right), a pool of cells expressing stable substrates was spiked-in to broaden the stability distribution of the library, followed by partitioning into six equal bins by FACS to enable measurement of the stability of each ORF–sgRNA pair. **c**,**d**, Summary of the screen results: the majority of screens identified *CUL3* as a significant hit (**c**); example results from successful screens, where both the 1-bin and 6-bin approaches concordantly identified the same BTB adaptor (**d**). **e**,**f**, Validation of the screen results: GAN was correctly identified as the BTB adaptor targeting keratins that we validated in a panel of individual experiments by flow cytometry (**e**), and KLHL15 targets ZNF511 as assayed by cycloheximide chase assays in control (sgAAVS1) versus KLHL15 knockout (sgKLHL15) cells (**f**). Immunoblot (IB) and flow cytometry experiments were performed twice with similar results. Source numerical data are available in Supplementary Tables 13–17; unprocessed blots are available in source data. Source data

**Extended Data Fig. 6:**
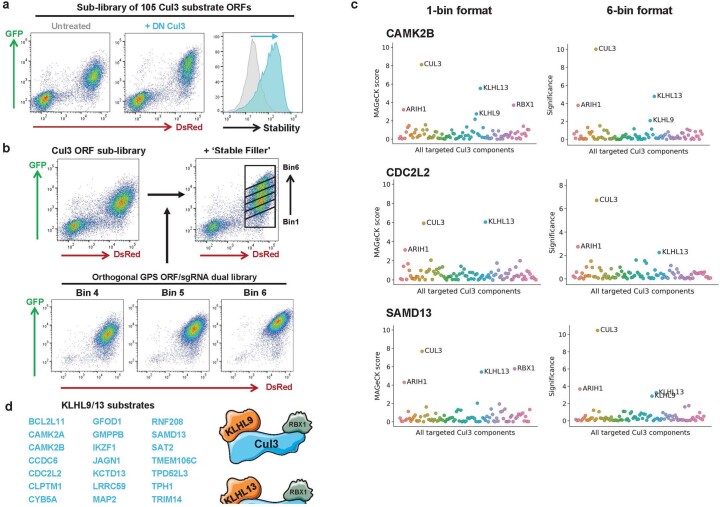
A multiplex CRISPR screen with full-length proteins targeted by Cul3 complexes. **a**, Validation of the Cul3 ORF sub-library. A sub-library of 116 ORFs was generated as in Extended Data Fig. 3c; the barcoded pool exhibited robust stabilization upon expression of dominant-negative Cul3. **b**, Broadening the stability distribution of the library for the screen in the ‘6-bin’ format. To enable the population to be sorted into 6 equal bins across the full stability spectrum, cells expressing orthogonal GPS ORF-sgRNA dual expression vectors in which the ORF substrates were stable were spiked-in at the appropriate ratio to yield an approximately even stability distribution. **c,d**, Example screen results for three substrates in which KLHL9 and/or KLHL13 (two paralogous BTB adaptors) were identified, together with a list of all putative KLHL9/13 substrates. Source numerical data are available in Supplementary Tables 13-17.

As the largest number of substrates were targeted by Cul3 complexes, we set out to identify the cognate BTB substrate adaptors responsible. We selected ~100 ORFs stabilized by DN Cul3 and cloned them into a barcoded GPS vector (Extended Data Fig. 5c) together with an sgRNA library targeting 95 Cul3 BTB adaptor proteins (4 sgRNAs per gene) to form the dual GPS/CRISPR multiplex screening library (Fig. 6a). For our initial multiplex screen with C-terminal peptides, all of the substrates exhibited roughly the same stability (Extended Data Fig. 1f). Here, however, the Cul3 substrates exhibited a much broader stability distribution (Extended Data Fig. 6a). To examine the optimal approach in this setting, we performed the multiplex screen in two different ways. In the 1-bin approach (Fig. 6b, left), we enriched for all stabilized substrates by sorting the top ~5% into a single tube. In the 6-bin approach (Fig. 6b, right), we first artificially broadened the stability of the library by spiking in a pool of cells expressing stable substrates (“stable filler”) to yield a more balanced stability distribution (Extended Data Fig. 6b). This allowed the population to be partitioned into six equal bins by FACS, allowing a stability measurement to be generated for each ORF–sgRNA combination (Fig. 6b, right).

Both multiplex screening approaches successfully identified *CUL3* as a significant hit in most of the screens: 90/111 (81%) using the 1-bin format, and 81/106 (76%) using the 6-bin format (Fig. 6c and Supplementary Tables 13–17). As a positive control, both sets of screens identified Gigaxonin (GAN, also known as KLHL16)—which is known to degrade a variety of intermediate filament proteins^21,22^—as the cognate BTB adaptor responsible for the degradation of Keratin (KRT)13, KRT15 and KRT16 (Fig. 6d,e). The screens also suggested relationships between KLHL8 and the mediator complex subunit MED27, and KLHL15 and the zinc finger protein ZNF511 (Fig. 6d,f). Furthermore, KLHL9 and/or KLHL13, two paralogous BTB adaptors sharing >90% identity, were identified as hits for multiple substrates (Extended Data Fig. 6c,d). Thus, multiplex CRISPR screening can be used to identify the cognate E3 ligases targeting full-length protein substrates and can be successful irrespective of the stability profile of the substrate pool.

### Multiplex CRISPR screening to define degron motifs

**Fig. 7:**
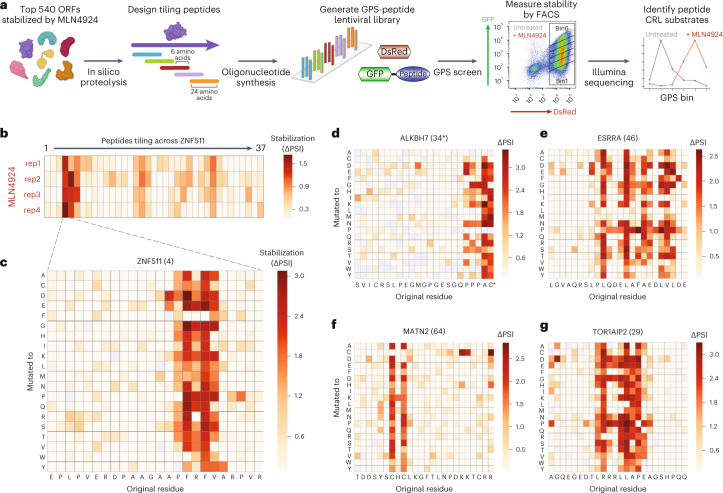
Systematic identification of linear motifs targeted by Cullin-RING E3 ubiquitin ligases. **a**–**c**, Schematic representation of the experimental strategy: a lentiviral GPS library of peptides substrates was generated through microarray oligonucleotide synthesis, wherein the same 540 ORFs exhibiting the greatest degree of stabilization upon MLN4924 treatment were expressed as a series of overlapping 24-mer tiles (**a**); comparative stability profiling in the presence and absence of MLN4924 then identified GFP–peptide fusions which were targeted by CRLs (**b**); and for the peptide substrates which exhibited the largest degree of stabilization, saturation mutagenesis was performed to quantify the stability of a panel of mutants in which each residue was mutated to all other possible residues, thereby defining degron motifs at amino acid resolution (**c**). **d**–**g**, Example degron motifs targeted by Cullin-RING E3 ligases. Saturation mutagenesis results for substrates derived from the C-terminus of ALKBH7 (**d**) and internal peptides derived from ESRRA (**e**), MATN2 (**f**) and TOR1AIP2 (**g**) are shown. Source numerical data are available in Supplementary Tables 18 and 19.

**Extended Data Fig. 7:**
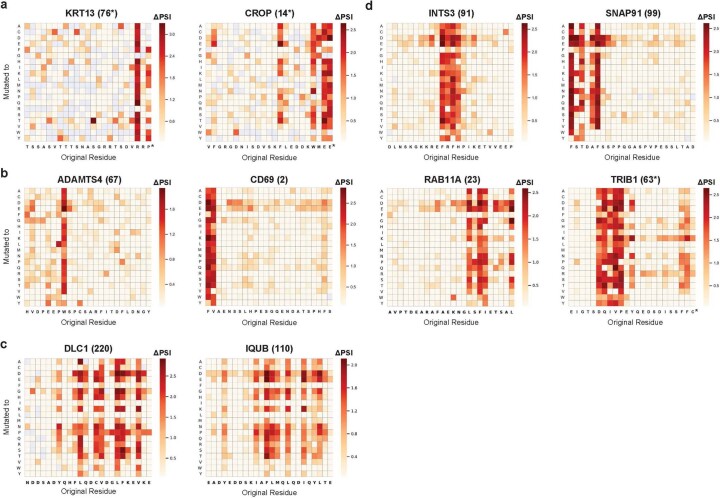
Linear degrons targeted by Cullin-RING E3 ligases. **a-d**, Example degron motifs delineated through site-saturation mutagenesis: **(a)** C-terminal degrons, **(b)** ‘narrow hydrophobic’ degrons, **(c)** ‘broad hydrophobic’ degrons, and **(d)** more complex degrons. Source numerical data are available in Supplementary Tables 18-19.

We reasoned that by combining multiplex CRISPR screening with saturation mutagenesis of peptide substrates, we could exploit the platform to define the degron motifs recognized by E3 ligases at scale. We started by mapping a set of degron motifs targeted by CRLs at amino acid resolution. We synthesized an oligonucleotide library encoding 24-mer peptides tiling across the leading 540 CRL substrate ORFs that we identified previously, cloned them into the lentiviral GPS vector downstream of GFP, and then performed an initial stability screen in the presence and absence of MLN4924 to define peptides harbouring degron motifs targeted by CRLs (Fig. 7a and Supplementary Table 18). For the peptides most strongly stabilized upon MLN4924 treatment, we then went on to perform saturation mutagenesis GPS screens, in which the stability of a panel of mutant versions of each peptide is measured; each amino acid is mutated to all other possible amino acids, thereby defining degron motifs at amino acid resolution (Fig. 7b,c and Supplementary Table 19). We identified multiple classes of degrons: C-terminal degrons (Fig. 7d and Extended Data Fig. 7a), the vast majority of which harboured known C-degron motifs^12^; hydrophobic degrons, ranging in size from seemingly individual tryptophan or phenylalanine residues up to a panel of hydrophobic amino acids spread across ten or more residues (Fig. 7e,f and Extended Data Fig. 7b,c); and a variety of more complex degrons, composed of a variety of amino acids and ranging from approximately four to eight consecutive amino acids in size (Fig. 7g and Extended Data Fig. 7d).

**Fig. 8:**
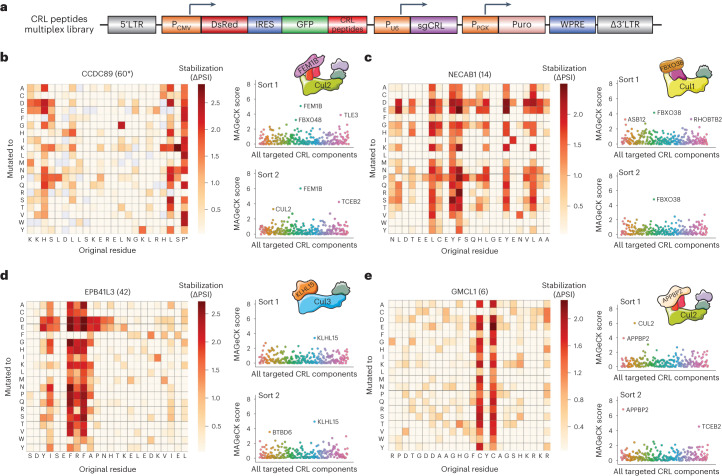
A multiplex CRISPR screen assigns Cullin-RING E3 ligases to their cognate degrons. **a**, Schematic representation of the multiplex CRISPR screening vector, wherein peptides with mapped degrons were fused to the C-terminus of GFP and the CRISPR sgRNA library targeted all known Cullin substrate adaptors. **b**–**e**, Assigning Cullin-RING E3 ligases to their cognate linear degrons. Data are shown for substrates derived from the C-terminus of CCDC89 (**b**) and internal peptides derived from NECAB1 (**c**), EPB41L3 (**d**) and GMCL1 (**e**): in each case, the saturation mutagenesis results mapping the degron motif are shown (left), alongside the multiplex CRISPR screen results after both one sort and two sorts (right). Source numerical data are available in Supplementary Tables 20–38.

**Extended Data Fig. 8:**
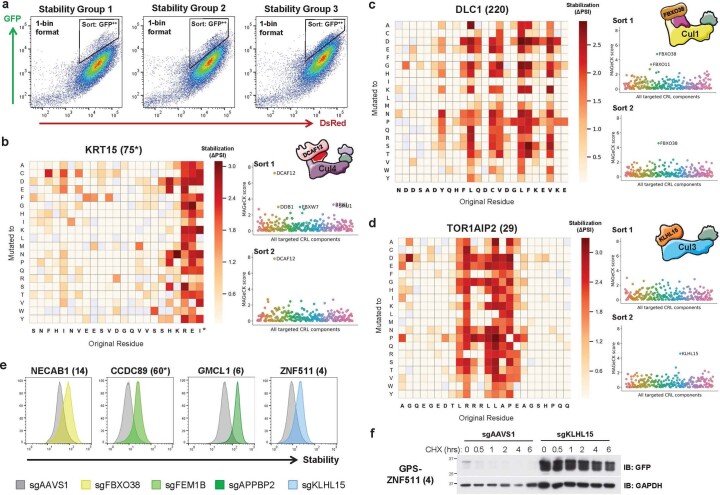
Assigning linear degron motifs to their cognate Cullin-RING E3 ligase. **a**, Sorting plots for the multiplex CRISPR screen. Three multiplex CRISPR screening libraries were generated in which substrates were divided into three groups based on their expected stability; in each case, the top ~5% of cells based on the stability of the GFP-peptide fusion were isolated by FACS. **b-d**, Assigning Cullin-RING substrate adaptors to cognate degron motifs: a C-terminal degron harboring an E-2 motif correctly assigned to DCAF12 **(b)**, a ‘broad hydrophobic’ degron assigned to FBXO38 **(c)**, and a complex degron assigned to KLHL15 **(d)**. **e,f**, Individual validation of the multiplex CRISPR screening results. **e**, The indicated peptide substrates expressed in the context of the GPS system were introduced into either control (sgAAVS1, gray) or knockout (yellow, green and blue) HEK-293T cells and their stability measured by flow cytometry. **f**, A cycloheximide chase assay was also used to monitor the degradation of the ZNF511 (4) peptide substrates in control (sgAAVS1) or KLHL15 knockout (sgKLHL15) cells by immunoblot. Immunoblot and flow cytometry experiments were performed twice with similar results. Source numerical data are available in Supplementary Tables 20-38; unprocessed blots are available in source data. Source data

We selected ~80 CRL peptide substrates harbouring degron motifs clearly defined by the saturation mutagenesis for multiplex CRISPR screening. We divided the substrates into three groups based on their stability (Extended Data Fig. 8a), and generated three dual GPS/CRISPR multiplex CRISPR screening libraries through the addition of a library of sgRNAs targeting 259 known CRL adaptors (4 sgRNAs per gene) (Fig. 8a). The screens were performed using the ‘1-bin’ approach, with the selected cells sorted twice: we anticipated that the earlier sort 1 would increase the likelihood of recovering potentially toxic mutations that would drop out later, while the subsequent sort 2 might deliver cleaner data owing to a purer population of selected cells (Supplementary Tables 20–37).

The efficacy of this approach was supported by the correct identification of the cognate adaptor for multiple positive control peptides harbouring C-terminal degrons: DCAF12 was identified as the CRL adaptor recognizing a C-terminal E-2 motif derived from the C-terminus of KRT15 (Extended Data Fig. 8b), and, further supporting the notion of a Pro/C-degron pathway regulated by FEM1B, FEM1B was identified as the CRL adaptor targeting a peptide derived from the C-terminus of CCDC89 terminating with a proline residue (Fig. 8b). Multiple broad hydrophobic degrons were found to be targeted by the Cul1 adaptor FBXO38 (Fig. 8c and Extended Data Fig. 8c), while the Cul3 adaptor KLHL15 was responsible for targeting several of the more complex degrons that mostly comprised F, R, L and P residues (Fig. 8d and Extended Data Fig. 8d); this is consistent with an “FRY” degron motif that has been previously characterized in two of its substrates, PP2A/B′β^23^ and CtIP^24^. We also identified APPBP2 as the cognate CRL adaptor responsible for recognition of a degron comprising twin cysteine residues (Fig. 8e). We validated a number of these E3 ligase–degron relationships identified by the screen in individual experiments (Extended Data Fig. 8e,f). Thus, the application of multiplex CRISPR screening to peptide substrates allows the identification of the cognate linear degrons recognized by E3 ligases.

## Discussion

While there are numerous high-throughput approaches for studying DNA and RNA biology on a systems-wide scale, similar approaches for studying protein stability are lacking. Here we combine our GPS expression screening system with loss-of-function CRISPR guide RNA libraries in a multiplex format, allowing for the high-throughput identification of E3 ligase–substrate pairs. In addition to identifying many previously studied degradative pathways, our multiplex technology provides insights into the substrate specificity for a panel of E3 ligases.

We focused our analysis on CRLs, a family of ~300 ubiquitin ligases that are critical mediators of signalling and of the response to cellular stressors^25^. Using a C-terminal peptide library enriched in CRL substrates, we were able to update our understanding of the C-degron pathways recognized by CRLs. First, we found that Cul4^DCAF12^ can recognize C-terminal peptides ending in -EI*, -EM* and -ES* in addition to the canonical twin-glutamic acid -EE* motif. Second, Cul4^TRPC4AP^ exhibits flexibility in its recognition of C-terminal arginine degrons, as it targets substrates with arginine at the −5 and −4 positions in addition to those with arginine at the −3 position. Third, Cul2^FEM1B^ can recognize C-terminal degrons ending in proline. A Pro/N-degron pathway was recently uncovered through which the GID E3 ligase complex targets N-terminal proline^26^, indicating that the same terminal residue can act as a degron at both the N-terminus and C-terminus. This is similar to glycine^11,12,27^ and arginine^12,16,17,18^, residues which can also act as both N-degrons and C-degrons^28^. Our results also highlight the flexibility of multiplex screening by identifying E3s for both full-length proteins and short peptides. This allowed us to identify a range of substrates, many of which previously unknown, recognized by Cul1^FBXO38^, Cul2^APPBP2^, Cul3^GAN^, Cul3^KLHL8^, Cul3^KLHL9/13^ and Cul3^KLHL15^.

Our mutagenesis experiments identified a wide variety of non-N/C-terminal degron motifs recognized by CRLs. Among the diversity of degrons are a variety of predominately hydrophobic motifs: a twin cysteine motif recognized by Cul2^APPBP2^, 3–5 hydrophobic residues recognized by Cul3^KLHL15^ and 8–12 hydrophobic residues across an ~20 residue span recognized by Cul1^FBXO38^. Although these hydrophobic motifs could have regulatory or signalling roles in certain contexts, we speculate that these degrons are unlikely to be accessible in the context of a folded protein and hence are likely to be exploited for quality control purposes. Indeed, exposed hydrophobicity is a feature often used by quality control pathways to recognize proteins that are unfolded, damaged or not paired with binding partners^29^. Consistent with this, AlphaFold predictions suggest that many of the hydrophobic degrons we identified are likely to exist in ordered structures when in their native context (Supplementary Table 18).

In some cases, we observed that a single E3 ubiquitin ligase can recognize multiple distinct degron motifs. The most prominent example is Cul2^FEM1B^, which controls the response to reductive stress by targeting FNIP1 for degradation through recognition of a cysteine-rich degron^15,30^. FEM1B has also been shown to recognize C-terminal arginine^12,16–18^. Here we show that FEM1B can additionally recognize substrates ending with proline in conjunction with internal aromatic residues often more than 15 amino acids away. Our analysis of these degrons using AlphaFold together with saturation mutagenesis data suggest that FEM1B has at least three regions for binding distinct motifs: C-termini ending in proline or arginine, single bulky hydrophobic residues, and cysteine- or histidine-rich sequences. In some cases, substrates need to engage two of these sites simultaneously for efficient recruitment to FEM1B. Furthermore, in an accompanying manuscript we identify a class of internal hydrophobic degrons which bind FEM1B by engaging the aromatic-binding pocket but not the Arg/Pro −1 pocket^31^. FEM1B is composed of multiple ankyrin and tetratricopeptide repeat domains, an architecture that may provide both the surface area and evolutionary flexibility to accommodate distinct degron-binding modes. Since many Cullin adaptors are composed of similar repeated domains, we speculate that the ability to recognize multiple different degrons is probably a shared property.

**Extended Data Fig. 9:**
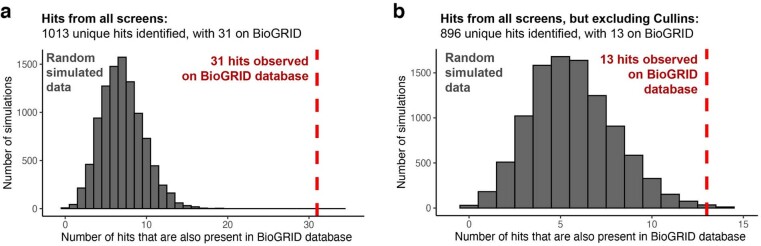
Comparison of multiplex CRISPR screen hits with physical interactions in BioGRID. **a**, Across all multiplex screens there were 1013 unique E3-substrate hits, of which 31 were present in the BioGRID interaction database. Datasets were simulated with 1013 hits chosen at random and we most frequently found that 7 of these hits were present in the BioGRID database. The 31 hits that we observe is therefore highly significant (p < 0.0001). **b**, The same analysis was performed as in (**a**) except that all E3-substrate hits that included a Cullin were excluded.

While multiplex screening can map E3–substrate interactions at higher throughput compared with proteomics, our approach does have some weaknesses. In our system, each substrate is overexpressed as an EGFP fusion that may not be behave in the same way as the endogenous protein. False negatives can also arise if there are multiple redundant E3s that target the same substrate, or if the CRISPR guides targeting the relevant E3 do not efficiently generate loss-of-function mutations. It is also possible that some of the E3 ligase–substrate relationships that we identified may not represent direct interactions, although our hits were enriched for physical interactions annotated in the BioGRID database^32^ (Extended Data Fig. 9 and Supplementary Table 38). Still, we believe that our multiplex approach is a valuable screening technique that can be used in conjunction with proteomics and biochemistry for elucidating degradative pathways.

Finally, many of the E3–substrate relationships that we describe may play important roles in human health. Mutations in the Cul3 adaptor GAN give rise to giant axonal neuropathy^22^ and heterozygous mutations in KLHL15 are associated with an intellectual development disorder^33,34^. Dominant mutations in FBXO38 cause spinal muscular atrophy^35^ and homozygous missense mutations cause distal hereditary motor neuronopathy^36^. We speculate that FBXO38 may play a role in the quality control of unfolded proteins, as the degron that it recognizes is predominantly hydrophobic. Finally, FEM1B mutations are associated with developmental delay and intellectual disability^37^. Thus, our mapping of degrons for KLHL15, FBXO38 and FEM1B may help guide the identification of substrates that aberrantly accumulate in the nervous system and give rise to disease.

## Methods

### Cell culture

HEK-293T (ATCC CRL-3216) cells were grown in Dulbecco’s modified Eagle medium (Life Technologies), which was supplemented with 10% foetal bovine serum (HyClone) and penicillin–streptomycin (Thermo Fisher Scientific).

### Antibodies and chemicals

Primary antibodies used were mouse M2 anti-FLAG (Sigma F3165; used at a dilution of 1:1,000), rabbit anti-β-actin (Cell Signaling 13E5; 1:10,000), mouse anti-GFP (Santa Cruz Biotech sc-9996; 1:1,000), rabbit anti-GAPDH (Cell Signaling D16H11; 1:10,000) and rabbit anti-Vinculin (Abcam ab129002; 1:10,000). Horseradish peroxidase (HRP)-conjugated goat anti-mouse/-rabbit secondary antibodies were obtained from Jackson ImmunoResearch (1:20,000) or Thermo Fisher Scientific (1:20,000). MLN4924 (used at 1 µM) was obtained from Active Biochem and cycloheximide from Calbiochem (100 µg ml^−1^).

### Lentivirus production

Lentivirus was packaged through the transfection of HEK-293T cells using PolyJet In Vitro DNA Transfection Reagent (SignaGen Laboratories). HEK-293T was seeded such that they reached ~80% confluency at the time of transfection. The transfection procedure recommended by the manufacturer was followed, with half of the DNA being the lentiviral transfer vector and the other half of the DNA comprising a mix of four plasmids encoding Gag-Pol, Rev, Tat and VSV-G. The medium was replaced with fresh Dulbecco’s modified Eagle medium 24 h post-transfection. Lentiviral supernatants were then collected at 48 h post-transfection, centrifuged (800*g*, 5 min) to pellet cell debris, and stored in single-use aliquots at −80 °C.

### Immunoblot

Cells were washed once in phosphate-buffered saline (PBS) and then lysed in 1% sodium dodecyl sulfate supplemented with 1:200 benzonase (Merck) for 20 min at room temperature. Lysates were heated to 70 °C for 10 min before separation by sodium dodecyl sulfate–polyacrylamide gel electrophoresis (mPAGE, Merck). Proteins were transferred to polyvinylidene difluoride (Immobilon-P, Merck) membrane (Trans-Blot SD Semi-Dry Transfer System, Bio-Rad). After blocking for 30 min in 5% skimmed milk (Sigma) dissolved in PBS, primary antibodies were applied overnight. Following three 5 min washes in PBS plus 0.2% Tween-20 (Sigma), HRP-conjugated secondary antibodies were applied for 40 min at room temperature. Reactive bands were visualized using Pierce ECL or Pico Western Blotting Substrate (Thermo Fisher Scientific) and a ChemiDoc Imaging System (Bio-Rad).

### Cycloheximide chase assays

Confluent 12-well plates of HEK-293Ts were treated with 100 µg ml^−1^ cycloheximide (Calbiochem). At the indicated time, cells were washed once with PBS and then directly lysed with NuPAGE LDS Sample Buffer (Thermo Fisher Scientific) supplemented with 50 mM dithiothreitol. Samples were sonicated for 20 s total using a probe sonicator (Thermo Fisher Scientific) and heated to 50 °C for 10 min before separation on 4–12% Bis-Tris gels (Thermo Fisher Scientific). Proteins were transferred to nitrocellulose using a Trans-Blot Cell (Bio-Rad). Membranes were blocked in 5% (w/v) skimmed milk (Thermo Fisher Scientific) dissolved in TBS-T (Tris-buffered saline with 0.1% Tween-20, Cell Signaling) and primary antibodies were applied overnight. Following three 5 min washes in TBS-T, HRP-conjugated secondary antibodies were applied for 1 h at room temperature. Reactive bands were visualized using Immobilon Western Chemiluminescent HRP Substrate (Millipore) and autoradiography film (Denville Scientific).

### Plasmids

An entry vector encoding FEM1B was obtained from the Ultimate ORFeome collection (Thermo Fisher Scientific) and transferred into a lentiviral destination vector encoding two N-terminal FLAG tags driven by the human cytomegalovirus (CMV) promoter through a Gateway LR reaction (Thermo Fisher Scientific). Point mutations were generated through the Gibson assembly (HiFi DNA Assembly Cloning Kit, NEB) of two overlapping fragments generated by PCR (Q5, NEB). Plasmids encoding C-terminally truncated DN Cullin constructs were a generous gift from Prof. Wade Harper; these were amplified by PCR and shuttled into a pHAGE lentiviral vector such that they also co-expressed blue fluorescent protein (BFP) downstream of a 2A peptide. Individual CRISPR/Cas9-mediated gene disruption experiments were performed using the lentiCRISPR v2 vector (Addgene #52961, deposited by Feng Zhang). The top and bottom strands of the sgRNAs were synthesized as oligonucleotides (IDT), phosphorylated using T4 PNK (NEB), annealed by heating to 95 °C followed by slow cooling to room temperature, and ligated (T4 ligase, NEB) into the lentiCRISPR v2 vector cut with BsmBI. Nucleotide sequences of the sgRNAs used were:

sg-AAVS1: GGGGCCACTAGGGACAGGAT

sg1-FEM1B: GTGACATAGCCAAGCAGATAG

sg2-FEM1B: GATGTACCTACCCGTCGAAG

sg-APPBP2: GATGTAGTTGTCCACGACAG

sg-GAN: GGTGCAGAAGAACATCCTGG

sg-FBXO38: GTTGTAGATCTCTGTGCAGGG

sg-KLHL15: GTCTGAAGTAATCACTCTGGG

### Flow cytometry

Flow cytometry analysis was performed on a BD LSRII instrument (Becton Dickinson). Cell sorting was performed on a MoFlo Astrios (Beckman Coulter). All data analysis was performed using FlowJo software.

### Multiplex CRISPR screen with C-terminal peptides

Dual substrate/sgRNA libraries for multiplex CRISPR screens were constructed by first generating a library of substrates fused to GFP in the context of the GPS lentiviral vector, followed by the addition of a downstream U6-sgRNA cassette encoding a library of CRISPR sgRNAs. To generate a substrate library enriched for C-terminal degrons, genomic DNA was extracted from cells harbouring lentiviral GPS vectors encoding GFP–peptide fusions stabilized by expression of DN Cul2 or DN Cul4 (Extended Data Fig. 1d). The peptides were amplified by PCR (Q5 Hot Start High-Fidelity DNA Polymerase, NEB) and cloned downstream of GFP into the lentiviral GPS vector cut with BstBI and XhoI using Gibson assembly (NEBuilder HiFi DNA Assembly Cloning Kit, NEB). Assembled products were purified and concentrated using SPRI beads (AMPure XP Reagent, Beckman Coulter), electroporated into DH10β cells (Thermo Fisher Scientific), and then grown overnight at 30 °C on Luria–Bertani (LB)-agar plates containing 100 µg ml^−1^ carbenicillin. The next morning all the resulting colonies were scraped from the plates and the plasmid DNA extracted (GenElute HP Plasmid DNA Midiprep Kit, Merck). Successful library construction was initially verified by Sanger sequencing (Azenta).

A custom sgRNA library targeting either Cul2/5 adaptors or Cul4 adaptors (six sgRNAs per gene) was synthesized as an oligonucleotide pool (Twist Bioscience), amplified by PCR (Q5 Hot Start High-Fidelity DNA Polymerase, NEB), purified (Qiagen PCR purification kit) and digested with BbsI (NEB). Following concentration by ethanol precipitation, the sample was separated on a 10% TBE polyacrylamide gel electrophoresis gel (Thermo Fisher Scientific) stained with SYBR Gold (Thermo Fisher Scientific) and the DNA was isolated from the 28 bp band using the ‘crush-and-soak’ method. The DNA was concentrated by ethanol precipitation and then cloned into lentiCRISPR v2 (Addgene #52961) digested with BsmBI (NEB). The U6-sgRNA cassette was then amplified by PCR, purified by agarose gel electrophoresis (QIAEX II Gel Extraction Kit, Qiagen), and cloned into the GPS-peptide substrate library plasmid pool linearized by digestion with I-SceI (NEB) using the Gibson assembly method (NEBuilder HiFi DNA Assembly Cloning Kit, NEB). At least 100-fold representation of the library was maintained at each step.

#### Multiplex CRISPR screening procedure

The dual GPS/sgRNA multiplex CRISPR screening plasmid library was packaged into lentiviral particles, which were used to transduce HEK-293T cells stably expressing Cas9 at a multiplicity of infection of ~0.2 (achieving approximately 20% DsRed^+^ cells) and at sufficient scale to achieve at least ~100-fold coverage of the library (number of GPS substrates × number of CRISPR sgRNAs × 100). Two days post-transduction, puromycin (1.5 µg ml^−1^) was added to eliminate untransduced cells. Surviving cells were pooled, expanded, and then at day 8 post-transduction partitioned by FACS into six equal bins based on the GFP/DsRed ratio.

Genomic DNA was extracted from both the selected cells and the unsorted library (Gentra Puregene Cell Kit, Qiagen), and the fusion peptides and associated sgRNAs were amplified by PCR (Herculase II Fusion Polymerase, Agilent) using a set of forward primers annealing between GFP and the fusion substrate and a set of reverse primers annealing to the tracrRNA downstream of the sgRNA. In each case a pool of eight primers were used, which differed from each other by one nucleotide in order to ‘stagger’ the resulting sequence reads to provide sufficient sequence diversity. In total, sufficient PCR reactions (4 µg genomic DNA in 100 µl) were performed to amplify a total amount of genomic DNA equivalent to the amount of genomic DNA from cells representing at least 100-fold coverage of library. All of the PCR reactions were pooled; approximately one-tenth was removed, purified using a spin column (Qiagen PCR purification kit), and 250 ng was used as a template for a second PCR reaction to add Illumina P5 and P7 adaptors and indexes. Indexed samples were then pooled to allow multiplexing, purified by agarose gel electrophoresis (QIAEX II Gel Extraction Kit, Qiagen) and sequenced using paired-end reads on either an Illumina NextSeq 550 or NovaSeq 600 instrument.

#### Multiplex CRISPR screen data analysis

Screens performed using the ‘1-bin’ format were analysed using the MAGeCK algorithm^13^. Constant sequences were removed from the raw Illumina reads using Cutadapt^38^ yielding a set of forward reads encoding the substrate and a set of reverse reads encoding the sgRNA. These were independently mapped to custom indexes using Bowtie 2 (ref. ^39^) and the resulting sam files combined such that each read was assigned to both a GPS substrate and associated sgRNA. For each individual GPS substrate, count tables were then generated enumerating how many times each sgRNA was identified in the unselected starting library compared with the sorted cells; these were subsequently analysed by MAGeCK to identify the genes targeted by sgRNAs enriched in the sorted cells. The MAGeCK output was visualized as a scatter plot using the Seaborn library, with all genes targeted arranged alphabetically on the *x* axis and the negative log_10_ of the MAGeCK ‘pos|score’ on the *y* axis. A step-by-step protocol is available at Protocol Exchange^40^.

### GPS-ORFeome screen

The generation of a GPS lentiviral vector expressing a barcoded human ORFeome was described previously^12^. The library was packaged into lentiviral particles and introduced into HEK-293T cells at a multiplicity of infection of ~0.2 (achieving approximately 20% DsRed^+^ cells) and at sufficient scale to achieve at least ~100-fold coverage of the library (~10 million transduced cells). Following puromycin selection (1.5 µg ml^−1^) to eliminate untransduced cells commencing 2 days post-transduction, cells were partitioned into six bins of equal size based on the stability of the GFP fusion (GFP/DsRed ratio). Control cells (dimethyl sulfoxide (DMSO)-treated) were sorted first, followed by cells treated with the pan-CRL inhibitor MLN4924 (1 µM for 8 h) using the identical gates and settings. Genomic DNA was then extracted (Gentra Puregene Cell Kit, Qiagen) from each of the sorted populations and Illumina sequencing libraries generated as described above, using primers binding in constant regions flanking the barcode cassette for the first PCR reaction, followed by a second PCR reaction to add Illumina indexes and P5 and P7 adaptors. Single-end sequencing was performed on a NextSeq 550 instrument (Illumina). Data analysis was performed as described previously^12^, yielding a protein stability index (PSI) stability metric between 1 (maximally unstable) and 6 (maximally stable) for each barcoded ORF. Candidate CRL substrates were identified by subtracting the PSI score in the DMSO treatment from the PSI score in the MLN4924 treatment, yielding a ΔPSI_MLN4924_ in each case.

### Generation of a barcoded sublibrary of MLN4924-responsive ORFs

Gateway entry vectors encoding each of the 540 ORFs were grown up individually from glycerol stocks in deep-well 96-well plates at 37 °C with vigorous shaking. The bacteria from each 96-well plate were then pooled evenly and the plasmid DNA extracted by miniprep (Qiagen). A Gateway LR reaction (Gateway LR Clonase II Enzyme mix, Thermo Fisher Scientific) was then performed (as per the manufacturer’s recommendations) to shuttle the ORFs into a GPS destination vector containing a random (22 N) ‘barcode’ sequence, such that, following column purification (Qiagen PCR purification kit) and transformation into DH10β cells (Thermo Fisher Scientific), the resulting recombinants expressed the ORFs as C-terminal fusions to GFP followed by a unique 3′ barcode. Sufficient colonies were scraped from the LB-agar plates to give an average of between four and five unique barcodes per ORF and the plasmid DNA extracted by midiprep (GenElute HP Plasmid DNA Midiprep Kit, Merck).

Barcodes were assigned to their corresponding upstream ORFs by paired-end Illumina sequencing. Plasmid DNA was first sheared (NEBNext dsDNA Fragmentase, NEB) to yield fragments with a mean size of ~500 bp, followed by end-repair and adaptor ligation according to the manufacturer’s protocol (NEBNext Ultra II DNA Library Prep Kit for Illumina, NEB). An initial PCR reaction was then performed using one primer annealing immediately downstream of the barcode and one primer binding the adaptor, thus enriching for fragments containing the barcode sequence on one end and a portion of the 3′ end of the upstream ORF on the other. Following a second PCR reaction to introduce Illumina P5 and P7 sequences, the products were sequenced on an Illumina MiSeq instrument using 150 bp paired-end reads: the forward reads were trimmed of constant sequence to reveal the sequence of the 22 nt barcode, while the reverse reads were mapped to a custom Bowtie 2 index composed of the 540 target ORFs to assign the associated ORF.

### GPS-ORFeome sublibrary screen with DN Cullins

The leading 540 ORFs exhibiting the greatest degree of stabilization upon MLN4924 treatment with further characterized using DN Cullin constructs. The barcoded GPS-ORF sublibrary was expressed in HEK-293T cells as described above. Six days post-transduction, the cells were divided across seven plates and transduced with lentiviral vectors encoding either DN Cul1, DN Cul2, DN Cul3, DN Cul4A, DN Cul4B, DN Cul5 or an empty vector as a control; these vectors also contained a downstream 2A-BFP cassette to identify transduced cells. The BFP^+^ cells in each individual pool were then partitioned into six stability bins by FACS and analysed as described above, yielding a PSI metric for each barcoded ORF across each of the conditions.

### Multiplex CRISPR screen with Cul3 substrate ORFs

A total of 116 ORFs identified as substrates of Cul3 complexes were selected for analysis by multiplex CRISPR screening. A barcoded GPS library of these 116 ORFs was created as described above. A pool of sgRNAs targeting 187 BTB adaptors at a depth of 6 sgRNAs/gene were synthesized on an oligonucleotide microarray (Agilent) and cloned into the lentiCRISPR v2 vector as described above. The U6-sgRNA cassette was then amplified by PCR, and cloned into the I-SceI site by Gibson assembly to generate the multiplex CRISPR screening library.

The screen performed in the ‘1-bin’ format was carried out exactly as described above: the library was packaged into lentiviral particles, introduced into Cas9-expressing HEK-293T cells at low multiplicity of infection, untransduced cells were removed through puromycin selection, and then the top 5% of cells based on the GFP/DsRed ratio were isolated by FACS. The screen performed in the ‘6-bin’ format was initially carried out in the same way, except that, after puromycin selection, ‘stable filler’ cells were spiked-in at the appropriate ratio (~30%) to generate a broad, even stability distribution. These ‘stable filler’ cells had previously been transduced with an orthogonal dual GPS-sgRNA expression library, and had been isolated by FACS on the basis of bright GFP fluorescence. The resulting population was then partitioned into six equal bins on the basis of the GFP/DsRed ratio by FACS, and deconvoluted by Illumina sequencing as described above.

The screen performed in the ‘1-bin’ format was analysed as described above. Screens performed using the ‘6-bin’ format were treated similarly initially, yielding for each of the six sorting bins a count table enumerating the frequency with which each substrate–sgRNA combination was observed. After normalization for sequencing depth, a PSI metric was calculated for each substrate–sgRNA combination, given by multiplying the proportion of reads in each bin by the bin number (1–6), thus generating a score ranging between 1 (maximally unstable) to 6 (maximally stable). To identify E3 ligases targeted by multiple sgRNAs that resulted in stabilization of the substrate, a set of Mann–Whitney *U* tests were performed; for each set of sgRNAs targeting the same E3 ligase, the mean PSI score of the substrate when paired with those sgRNAs was compared with the mean PSI score for the substrate when paired with all other sgRNAs. The results were again visualized as a scatter plot, with all genes targeted arranged alphabetically on the *x* axis and the negative log_10_ of the resulting *P* value on the *y* axis.

One weakness of the 1-bin approach is that substrates lying at the bottom of the stability group will be placed at a disadvantage: upon knockout of the cognate E3, any degree of stabilization of substrates at the top of the stability group should be sufficient to shift the cells into the sorting gate, whereas for substrates at the bottom of the stability group a larger degree of stabilization will be required. Indeed, for our multiplex CRISPR screen with CRL degron peptides (Fig. 8), >75% of the substrates for which we obtained significant hits were predicted to lie in the top half of their stability group. Thus we would consider the 6-bin format optimal for future experiments, with that caveat that they are more complex to establish due to the requirement to balance the overall stability distribution of the substrates. However, the 1-bin format does allow for the possibility of a second sort to further purify the population of cells expressing stabilized GFP-fusion substrates before sequencing, and indeed we found that the data from the second sort were generally superior to the first (Fig. 8).

### GPS-peptide screen

Nucleotide sequences encoding a series of 24-mer peptide tiles starting at 6-mer intervals across the 540 ORFs (a total of 33,566 sequences) were synthesized on an oligonucleotide microarray (Agilent), amplified by PCR, and cloned into a lentiviral GPS vector downstream of GFP by Gibson assembly. To avoid the generation of C-terminal degrons a common C-terminal sequence (encoding the 10-mer RIARAKASTN*) was appended to all peptides, except for those peptides that were derived from the native C-terminus of the proteins that retained their stop codon at the native position. The GPS-peptide library was expressed in HEK-293T cells and the stability of the GFP–peptide fusions in the presence and absence of MLN4924 measured by FACS and Illumina sequencing as described above.

For the leading 791 peptides that exhibited both significant and reproducible responses to MLN4924 treatment, we performed saturation mutagenesis GPS screens to characterize the degron motif. Oligonucleotide libraries were synthesized (Agilent) encoding both the wild-type peptide plus a panel of single mutant variants in which each residue was mutated to all other possible residues. Following PCR amplification and cloning into the GPS vector downstream of GFP, the resulting GPS-peptide saturation mutagenesis library was expressed in HEK-293T cells and the stability of the GFP–peptide fusions measured by FACS and Illumina sequencing as described above. The results are depicted as heat maps, in which the colour of each cell illustrates the stability difference (ΔPSI) between that individual mutant peptide and the median PSI of all the unmutated peptides; the darker the red colour, the greater the stabilizing effect of the mutation.

### Multiplex CRISPR screen with Cullin-substrate peptides

Sixty-three peptide substrates with well-resolved degron motifs were selected for analysis by multiplex CRISPR screening. The peptides substrates were divided into three pools of equal size based on their stability, synthesized as oligonucleotides (Agilent) and cloned into the GPS vector downstream of GFP. An sgRNA library targeting known Cullin adaptors (259 genes at a depth of 4 sgRNAs per gene) was synthesized (Agilent) and cloned into lentiCRISPR v2 as described above; the U6-sgRNA cassette was then amplified by PCR and cloned into the GPS vector using the I-SceI site to generate the multiplex CRISPR screening library. Screens were performed in the 1-bin format as described above.

### AlphaFold and phylogenetic analysis of FEM1B

We predicted ten structures of FEM1B bound to different substrates using the AlphaFold plugin in ChimeraX (v1.4). The full peptide sequence and the full FEM1B sequence were used as inputs. Structural analysis and structural alignments were also performed in ChimeraX, with the Arg/Pro −1 pocket and aromatic-binding pocket residues defined on the basis of their predicted contact (van der Waals overlap ≥−0.70 Å) with the substrate proline or aromatic residues, respectively. Twelve FEM1B orthologues from diverse animal species were collected: *Homo sapiens* (Q9UK73), *Bos taurus* (F1N162), *Anolis carolinensis* (XP_003227293.1), *Mus musculus* (Q9Z2G0), *Gallus gallus* (Q5ZM55), *Drosophila melanogaster* (A1ZBY1), *Nematostella vectensis* (XP_001622320.2), *Danio rerio* (E7F7Y4), *Xenopus laevis* (Q6GPE5), *Anopheles gambiae* (A0A1S4GUZ4), *Apis mellifera* (XP_026298620.1) and *Ciona intestinalis* (XP_002128243.1). Sequences were aligned in Clustal Omega and visualized using ESPrint 3.

### Saturation mutagenesis of FEM1B peptide substrates

An oligonucleotide library was synthesized (Agilent) encoding both the wild-type peptide plus a panel of single mutant variants in which each residue was mutated to all other possible residues. In addition, an extra set of peptides were also encoded in which single additions of all 20 amino acids (labelled ‘Add’) were appended to the extreme C-terminus. GPS-peptide libraries were generated GPS screens performed to measure the stability of each mutant as described above. The results are depicted as heat maps, in which the colour of each cell illustrates the stability difference (ΔPSI) between that individual mutant peptide and the median PSI of all the unmutated peptides; the darker the red colour, the greater the stabilizing effect of the mutation.

### Comparison of multiplex screen data to BioGRID

A custom R script using the packages dplyr, ggplot2 and stringr was used to compare screen data hits to physical interactions on the BioGRID database. We used the Homo sapiens BIOGRID-4.4.220 release for our analysis. Briefly, we calculated for a screen containing random hits how many of these hits were also found on the BioGRID database. This process was then repeated for 10,000 random screens and compared to how many hits we found in common for our experimental multiplex data.

### Statistics and reproducibility

Unless specified in the legends, all screens were performed only once. Follow-up immunoblot and flow cytometry experiments were performed two independent times with similar results. No statistical methods were used to pre-determine sample size. No data were excluded from the analyses unless specified. Experiments were not randomized. The investigators were not blinded to allocation during experiments and outcome assessment.

### Reporting summary

Further information on research design is available in the Nature Portfolio Reporting Summary linked to this article.

## Online content

Any methods, additional references, Nature Portfolio reporting summaries, source data, extended data, supplementary information, acknowledgements, peer review information; details of author contributions and competing interests; and statements of data and code availability are available at 10.1038/s41556-023-01229-2.

### Supplementary information

Reporting Summary

Supplementary Tables 1–38 **Supplementary Tables 1–6 A multiplex CRISPR screen reveals C-terminal degrons targeted by Cul2 and Cul4 E3 ligase complexes.** MAGeCK output detailing all significant E3 ligase–substrate interactions identified from the Cul2 (Supplementary Table 1) and Cul4 (Supplementary Table 5) multiplex CRISPR screens. Substrates containing a motif known to be targeted by the substrate adaptor identified are indicated in the final column. The raw sequence read counts for each substrate–sgRNA combination are given in Supplementary Table 2 (Cul2 screen) and Supplementary Table 5 (Cul4 screen), together with the associated MAGeCK output for each substrate in Supplementary Table 3 (Cul2 screen) and Supplementary Table 6 (Cul4 screen). **Table 7 Saturation mutagenesis of FEM1B peptide substrates terminating with proline.** Stability profiling data for each mutant construct is given, showing the corrected read counts across the six stability bins. A PSI score was calculated for each mutant, which was compared with the median PSI of all the wild-type sequences to generate a ΔPSI stability metric reflecting the impact of the mutation. **Tables 8–10 Identification of Cullin-RING E3 ligases substrates through a GPS-ORFeome screen.** Stability profiling data showing the corrected read counts across the six stability bins in control cells (DMSO) or cells treated with the pan-Cullin inhibitor MLN4924. A PSI score was calculated for each ORF in each condition, generating a ΔPSI metric reflecting the degree of stabilization upon MLN4924 treatment. Stability profiling data for each ORF stabilized greater than 0.5 PSI units upon treatment with the pan-Cullin inhibitor MLN4924 are presented in Supplementary Table 8. Where there are multiple isoforms of a gene encoded in the library that cannot be uniquely distinguished by their associated barcodes (indicated by the lowercase ‘ioh’ designation), the sequence reads were allocated to all isoforms. The full dataset aggregated per ORF is presented in Supplementary Table 9, with the data for each individual barcoded ORF presented in Supplementary Table 10. **Tables 11 and 12 Assigning Cullin ORF substrates to their cognate CRL complex.** GPS stability profiling of a sublibrary of ORFs stabilized by MLN4924 was performed using DN Cullin constructs. Stability profiling data for each ORF assigned to a particular CRL complex are given in Supplementary Table 11, showing the corrected read counts across the six stability bins in control cells (‘EmptyVector’) or cells transduced with DNCul1 through DNCul5. A PSI score was calculated for each ORF in each condition, generating a ΔPSI metric reflecting the degree of stabilization upon inhibition of each CRL complex. The full dataset for all ORFs is provided in Supplementary Table 12. **Tables 13–17 A multiplex CRISPR screen reveals BTB adaptors recognizing full-length protein substrates targeted by Cul3 complexes.** Significant E3 ligase–substrate interactions from the screen performed in the ‘1-bin’ format (MAGeCK output) are presented in Supplementary Table 13, and the 6-bin format in Supplementary Table 14. For the screen performed in the ‘1-bin’ format, the raw sequence read counts for each substrate-sgRNA combination are given in Supplementary Table 15, together with the associated MAGeCK output for each substrate Supplementary Table 16. For the screen performed in the ‘6-bin’ format, stability profiling data showing the corrected read counts across the six stability bins are presented in Supplementary Table 17. This yielded a PSI score for each substrate–sgRNA combination, which was compared with the mean PSI score for the substrate. **Table 18 A GPS-peptide screen to identify peptide substrates targeted by Cullin-RING E3 ligases.** Stability profiling data showing the corrected read counts across the six stability bins for each peptide substrate in control (DMSO) versus MLN4924-treated cells. This yielded a PSI score for each peptide substrate in each condition, which was used to generate a ΔPSI stability metric reflecting the degree of stabilization upon MLN4924 treatment. **Table 19 Saturation mutagenesis to define degron motifs targeted by Cullin-RING E3 ligases.** Stability profiling data for each mutant construct are given, showing the corrected read counts across the six stability bins. A PSI score was calculated for each mutant, which was compared with the median PSI of all the wild-type sequences to generate a ΔPSI stability metric reflecting the impact of the mutation. **Tables 20–37 A multiplex CRISPR matches degrons to their cognate Cullin-RING E3 ligase.** MAGeCK output detailing all significant E3 ligase–substrate interactions identified from each of the screens is presented in Supplementary Table 20 (group 1, sort 1), Supplementary Table 21 (group 1, sort 2), Supplementary Table 22 (group 2, sort 1), Supplementary Table 23 (group 2, sort 2), Supplementary Table 24 (group 3, sort 1) and Supplementary Table 25 (group 3, sort 2). The raw sequence read counts for each substrate–sgRNA combination are given in Supplementary Tables 26–31, together with the associated MAGeCK output for each substrate in Supplementary Tables 32–37. **Table 38 Overlap between putative E3 ligase–substrate relationships identified by multiplex CRISPR screening and physical interactions reported in BioGRID.**

### Source data

Source Data Figs. 3 and 6, and Extended Data Figs. 2, 4 and 8 Uncropped blot images of Figs. 3 and 6, and Extended Data Figs. 2, 4 and 8.

## Figures and Tables

**Extended Data Fig. 10:**
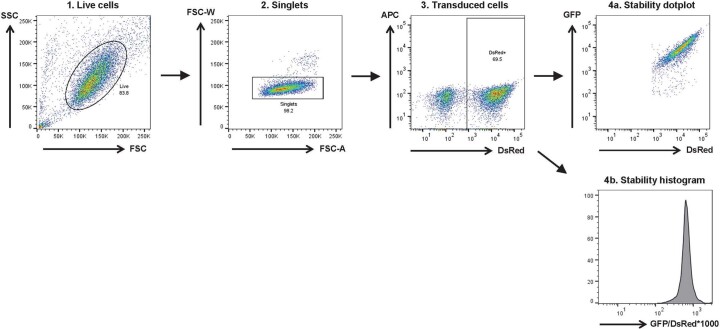
Representative FACS gating strategy for all GPS experiments.

## Data Availability

Sequencing data that support the findings of this study have been deposited in the Sequence Read Archive (SRA) under accession code PRJNA1001958. Both raw and processed data for all screens are provided in the supplementary tables. All other data supporting the findings of this study are available from the corresponding author on reasonable request. Source data are provided with this paper.

## Code availability

Sample Python code to analyse multiplex CRISPR screening sequencing data is available at https://github.com/rttimms/multiplex_CRISPR_screens. The R code to compare the multiplex screening hits to BioGRID data is available at https://github.com/elijahmena/biogridanalysis.
