# Author Correction: Defining E3 ligase–substrate relationships through multiplex CRISPR screening

**DOI:** 10.1038/s41556-023-01336-0

**Published:** 2023-12-19

**Authors:** Richard T. Timms, Elijah L. Mena, Yumei Leng, Mamie Z. Li, Iva A. Tchasovnikarova, Itay Koren, Stephen J. Elledge

**Affiliations:** 1 Department of Genetics, Harvard Medical School, Division of Genetics, Brigham asnd Women’s Hospital, Howard Hughes Medical Institute, Boston, MA USA; 2 Cambridge Institute of Therapeutic Immunology and Infectious Disease, Department of Medicine, University of Cambridge, Cambridge, UK; 3 Wellcome/CRUK Gurdon Institute, Department of Biochemistry, University of Cambridge, Cambridge, UK; 4 The Mina and Everard Goodman Faculty of Life Sciences, Bar-Ilan University, Ramat-Gan, Israel

**Keywords:** High-throughput screening, Ubiquitin ligases, CRISPR-Cas9 genome editing

Correction to: *Nature Cell Biology* https://www.nature.com/articles/s41556-023-01229-2, published online 21 September 2023.

It has been brought to the authors’ attention that the schematics in Fig. 5b were not described sufficiently clearly in the associated legend. The authors wish to clarify that the two identical flow cytometry plots shown in Fig. 5b depict a schematic representation of the screening process, and do not represent data from the screen. The experiment depicted in the schematic in Fig. 5b yielded data shown in Fig. 5c. The corrected Fig. 5b legend now reads, in part, “schematic representation of the comparative stability profiling screen using the pan-Cullin inhibitor MLN4924…,” as amended in the HTML and PDF versions of the article.

