# Supplementary material for: Defining E3 ligase–substrate relationships through multiplex CRISPR screening
Source: Nat Cell Biol. 2023 Sep 21;25(10):1535–45. doi: 10.1038/s41556-023-01229-2 (PMC10567573; doi:10.1038/s41556-023-01229-2)
Supplement: Supplementary file 1 — Reporting Summary [file 41556_2023_1229_MOESM1_ESM.pdf]

nature portfolio

Corresponding author(s): Elledge, SE

Last updated by author(s): Aug 7, 2023

Reporting Summary

Nature Portfolio wishes to improve the reproducibility of the work that we publish. This form provides structure for consistency and transparency in reporting. For further information on Nature Portfolio policies, see our Editorial Policies and the Editorial Policy Checklist.

Statistics

For all statistical analyses, confirm that the following items are present in the figure legend, table legend, main text, or Methods section.

n/a	Confirmed	
☐	☒	The exact sample size (*n*) for each experimental group/condition, given as a discrete number and unit of measurement
☐	☒	A statement on whether measurements were taken from distinct samples or whether the same sample was measured repeatedly
☐	☒	The statistical test(s) used AND whether they are one- or two-sided<br>*Only common tests should be described solely by name; describe more complex techniques in the Methods section.*
☒	☐	A description of all covariates tested
☒	☐	A description of any assumptions or corrections, such as tests of normality and adjustment for multiple comparisons
☒	☐	A full description of the statistical parameters including central tendency (e.g. means) or other basic estimates (e.g. regression coefficient) AND variation (e.g. standard deviation) or associated estimates of uncertainty (e.g. confidence intervals)
☒	☐	For null hypothesis testing, the test statistic (e.g. *F*, *t*, *r*) with confidence intervals, effect sizes, degrees of freedom and *P* value noted<br>*Give P values as exact values whenever suitable.*
☒	☐	For Bayesian analysis, information on the choice of priors and Markov chain Monte Carlo settings
☒	☐	For hierarchical and complex designs, identification of the appropriate level for tests and full reporting of outcomes
☒	☐	Estimates of effect sizes (e.g. Cohen's *d*, Pearson's *r*), indicating how they were calculated

Our web collection on statistics for biologists contains articles on many of the points above.

Software and code

Policy information about availability of computer code

Data collection	Flow cytometry data was collected using BD FACSDiva (8.0.3).
Data analysis	Analysis of multiplex screen data used Cutadapt (v4.1), Bowtie 2 (2.4.1), and the MAGeCK algorithm (0.5.9). A sample script to analyze multiplex CRISPR screening sequencing data is provided as Supplementary Code. Data visualization was performed using the Python Matplotlib (3.6.0) and Seaborn (v0.12) packages. BioGRID analysis was performed using the dplyr (1.1.0), ggplot2 (3.4.1), and stringr (1.5.0) R packages. Analysis of the FEM1B degron interaction used ChimeraX-1.4 (including its AlphaFold plugin), Clustal Omega, and ESPrint 3. Flow cytometry data was analyzed using FlowJo (10.4).

For manuscripts utilizing custom algorithms or software that are central to the research but not yet described in published literature, software must be made available to editors and reviewers. We strongly encourage code deposition in a community repository (e.g. GitHub). See the Nature Portfolio guidelines for submitting code & software for further information.

Data

Policy information about availability of data

All manuscripts must include a data availability statement. This statement should provide the following information, where applicable:

- Accession codes, unique identifiers, or web links for publicly available datasets
- A description of any restrictions on data availability
- For clinical datasets or third party data, please ensure that the statement adheres to our policy

Sequencing data that support the findings of this study have been deposited in the Sequence Read Archive (SRA) under accession code PRJNA1001958. Both raw and processed data for all screens is provided in the Supplementary Tables. All other data supporting the findings of this study are available from the corresponding author on reasonable request.

Research involving human participants, their data, or biological material

Policy information about studies with human participants or human data. See also policy information about sex, gender (identity/presentation), and sexual orientation and race, ethnicity and racism.

Reporting on sex and gender	N/A
Reporting on race, ethnicity, or other socially relevant groupings	N/A
Population characteristics	N/A
Recruitment	N/A
Ethics oversight	N/A

Note that full information on the approval of the study protocol must also be provided in the manuscript.

Field-specific reporting

Please select the one below that is the best fit for your research. If you are not sure, read the appropriate sections before making your selection.

☒ Life sciences ☐ Behavioural & social sciences ☐ Ecological, evolutionary & environmental sciences

For a reference copy of the document with all sections, see nature.com/documents/nr-reporting-summary-flat.pdf

Life sciences study design

All studies must disclose on these points even when the disclosure is negative.

Sample size	No sample size calculations were performed. All stages of multiplex CRISPR screening experiments were performed at sufficient scale to maintain at least 100-fold representation of the library and a minimum of 10,000 live cells were analyzed for flow cytometry experiments, as these are used as standard in the field.
Data exclusions	No data were excluded.
Replication	All screens were typically performed only once; however, all critical findings from the multiplex CRISPR screens were validated through individual CRISPR/Cas9 knockout experiments, and, in all cases tested, the screen results were replicated. For all other experiments, the data shown are typically representative of multiple independent experiments.
Randomization	The study design did not permit randomization to be employed. However, lentiviral particles encoding different library members integrate randomly, and thus which cells receive each substrate-guide combination is entirely random.
Blinding	Blinding was not necessary as we were unaware of the identity of hits during data collection and analysis.

Reporting for specific materials, systems and methods

We require information from authors about some types of materials, experimental systems and methods used in many studies. Here, indicate whether each material, system or method listed is relevant to your study. If you are not sure if a list item applies to your research, read the appropriate section before selecting a response.

Materials & experimental systems

n/a	Involved in the study	
☐	☒	Antibodies
☐	☒	Eukaryotic cell lines
☒	☐	Palaeontology and archaeology
☒	☐	Animals and other organisms
☒	☐	Clinical data
☒	☐	Dual use research of concern
☒	☐	Plants

Methods

n/a	Involved in the study	
☒	☐	ChIP-seq
☐	☒	Flow cytometry
☒	☐	MRI-based neuroimaging

Antibodies

Antibodies used	The following antibodies were used for immunoblot:<br>mouse M2 anti-FLAG (Sigma F3165; used at a dilution of 1:1000)<br>rabbit anti-β-actin (Cell Signaling 13E5; 1:10,000)<br>mouse anti-GFP (Santa Cruz Biotech sc-9996; 1:1,000)<br>rabbit anti-GAPDH (Cell Signaling D16H11; 1:10,000)<br>rabbit anti-Vinculin (Abcam ab129002; 1:10,000)
Validation	Validation data for all the above antibodies can be found at the following manufacturer websites:<br>https://www.sigmaaldrich.com/GB/en/product/sigma/f3165<br>https://www.cellsignal.com/products/primary-antibodies/b-actin-13e5-rabbit-mab/4970<br>https://www.scbt.com/p/gfp-antibody-b-2<br>https://www.cellsignal.com/products/primary-antibodies/gapdh-d16h11-xp-rabbit-mab/5174<br>https://www.abcam.com/products/primary-antibodies/vinculin-antibody-epr8185-ab129002.html

Eukaryotic cell lines

Policy information about cell lines and Sex and Gender in Research

Cell line source(s)	HEK293T cells were obtained from ATCC.
Authentication	Authenticated HEK293T cells were obtained from ATCC; we did not perform any additional authentication.
Mycoplasma contamination	Cells were routinely tested for Mycoplasma contamination and found to be negative.
Commonly misidentified lines<br>(See ICLAC register)	No commonly misidentified cell lines were used.

Flow Cytometry

Plots

Confirm that:

☒ The axis labels state the marker and fluorochrome used (e.g. CD4-FITC).

☒ The axis scales are clearly visible. Include numbers along axes only for bottom left plot of group (a 'group' is an analysis of identical markers).

☒ All plots are contour plots with outliers or pseudocolor plots.

☒ A numerical value for number of cells or percentage (with statistics) is provided.

Methodology

Sample preparation	HEK293T cells were trypsinized, washed once with PBS, and aliquoted into 5 ml FACS tubes.
Instrument	BD LSR II
Software	Data was collected using FACS DIVA and analyzed using FlowJo.
Cell population abundance	Live HEK293T cells were gated based on forward and side scatter and typically represented >80% of all events.
Gating strategy	For GPS experiments, gating for DsRed+ cells ensured that only transduced cells were analyzed.

☒ Tick this box to confirm that a figure exemplifying the gating strategy is provided in the Supplementary Information.
