# Supplementary figures and images for: Defining E3 ligase–substrate relationships through multiplex CRISPR screening
Source: Nat Cell Biol. 2023 Sep 21;25(10):1535–45. doi: 10.1038/s41556-023-01229-2 (PMC10567573; doi:10.1038/s41556-023-01229-2)

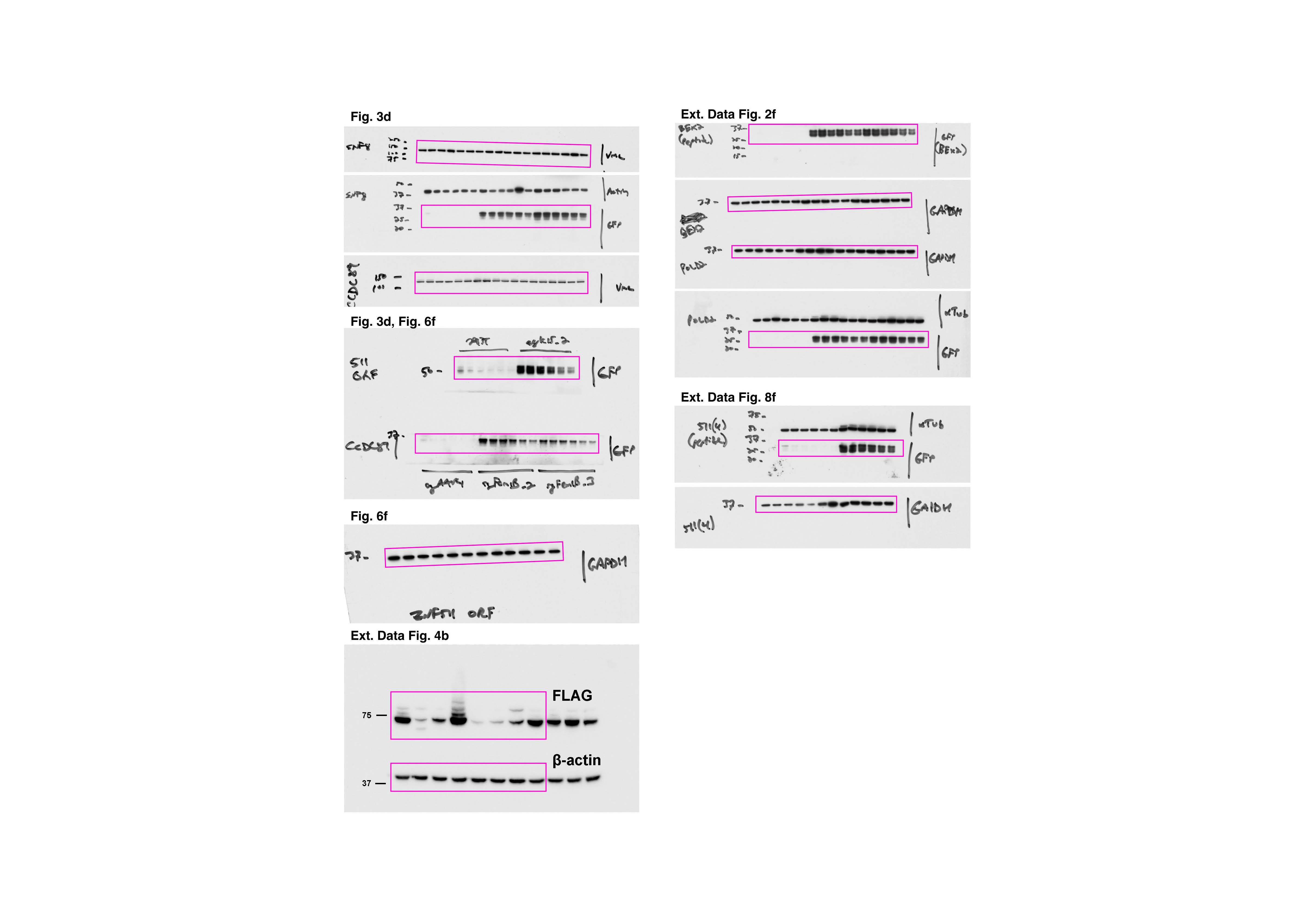

Supplement: Supplementary file 3 — Uncropped blot images of Figs. 3 and 6, and Extended Data Figs. 2, 4 and 8. [file 41556_2023_1229_MOESM3_ESM.jpg]
